# Anatomy and Cranial Functional Morphology of the Small-Bodied Dinosaur *Fruitadens haagarorum* from the Upper Jurassic of the USA

**DOI:** 10.1371/journal.pone.0031556

**Published:** 2012-04-11

**Authors:** Richard J. Butler, Laura B. Porro, Peter M. Galton, Luis M. Chiappe

**Affiliations:** 1 Bayerische Staatssammlung für Paläontologie und Geologie, Munich, Germany; 2 GeoBio-Center, Ludwig-Maximilians-Universität München, Munich, Germany; 3 Department of Organismal Biology and Anatomy, University of Chicago, Illinois, United States of America; 4 Professor Emeritus, University of Bridgeport, Bridgeport, Connecticut, United States of America; 5 Rio Vista, California, United States of America; 6 The Dinosaur Institute, Natural History Museum of Los Angeles County, Los Angeles, California, United States of America; Raymond M. Alf Museum of Paleontology, United States of America

## Abstract

**Background:**

Heterodontosaurids are an important but enigmatic and poorly understood early radiation of ornithischian dinosaurs. The late-surviving heterodontosaurid *Fruitadens haagarorum* from the Late Jurassic (early Tithonian) Morrison Formation of the western USA is represented by remains of several small (<1 metre total body length, <1 kg body mass) individuals that include well-preserved but incomplete cranial and postcranial material. *Fruitadens* is hypothesized to represent one of the smallest known ornithischian dinosaurs.

**Methodology/Principal Findings:**

We describe the cranial and postcranial anatomy of *Fruitadens* in detail, providing comparisons to all other known heterodontosaurid taxa. High resolution micro-CT data provides new insights into tooth replacement and the internal anatomy of the tooth-bearing bones. Moreover, we provide a preliminary functional analysis of the skull of late-surviving heterodontosaurids, discuss the implications of *Fruitadens* for current understanding of heterodontosaurid monophyly, and briefly review the evolution and biogeography of heterodontosaurids.

**Conclusions/Significance:**

The validity of *Fruitadens* is supported by multiple unique characters of the dentition and hindlimb as well as a distinct character combination. *Fruitadens* shares highly distinctive appendicular characters with other heterodontosaurids, strengthening monophyly of the clade on the basis of the postcranium. Mandibular morphology and muscle moment arms suggest that the jaws of late-surviving heterodontosaurids, including *Fruitadens*, were adapted for rapid biting at large gape angles, contrasting with the jaws of the stratigraphically older *Heterodontosaurus*, which were better suited for strong jaw adduction at small gapes. The lack of wear facets and plesiomorphic dentition suggest that *Fruitadens* used orthal jaw movements and employed simple puncture-crushing to process food. In combination with its small body size, these results suggest that *Fruitadens* was an ecological generalist, consuming select plant material and possibly insects or other invertebrates.

## Introduction

Ornithischian dinosaurs were one of the most important groups of Mesozoic archosaurs, dominating the herbivorous macrofauna of the northern hemisphere during the Cretaceous [1]–[3]. The earliest ornithischians date from the Late Triassic of Argentina and South Africa [4]–[9], but they remained minor components of most terrestrial ecosystems during the first 70 million years of their evolution before radiating extensively during the Late Jurassic and Early Cretaceous [1]–[3]. One important clade of early ornithischians is the enigmatic and poorly understood Heterodontosauridae, a group characterized by an unusual and strongly heterodont dentition [10]–[14]. Heterodontosaurids are best known from the Lower Jurassic upper Elliot and Clarens formations of South Africa and Lesotho, with approximately 20 specimens (many of which remain incompletely studied) known from these strata [10]–[29]. These specimens form the basis for five monospecific genera, of which three (*Heterodontosaurus tucki*, *Abrictosaurus consors*, *Lycorhinus angustidens*) are considered valid in recent reviews [12]–[14], although a fourth taxon also appears to be present, and “*Lanasaurus scalpridens*" (double quotation marks indicate that a species may not be diagnostic, or that a proposed taxonomic grouping is probably non-monophyletic) may or may not be valid [14], [29]. The best represented taxon, *Heterodontosaurus tucki*, is known from two well-preserved skulls, one of which is associated with a nearly complete, articulated postcranium [10], [11], [14], [18], [22], as well as a partial juvenile skull [14], [27] and a fragmentary skull that is the largest known for any heterodontosaurid [29]. Other heterodontosaurids include *Tianyulong confuciusi*, known from a single exceptionally preserved specimen that preserves filamentous integumentary structures from the Middle–Late Jurassic of China [30] (previously reported as late Early Cretaceous, but see Lü *et al.* [31]), the historical taxon *Echinodon becklesii* from the earliest Cretaceous of the UK [1], [13], [32]–[39], a jaw fragment from the Late Triassic of Argentina [6], an undescribed specimen from the Early Jurassic of the USA [1], [40], the recently described *Manidens condorensis* from the Middle Jurassic of Argentina [41], and possibly the oldest known ornithischian, *Pisanosaurus mertii*, from the Late Triassic (late Carnian: see Martinez *et al.* [42]) of Argentina [5], [8], [12], [13]. Specimens previously assigned to Heterodontosauridae from the Late Triassic of Switzerland [43], the Early Jurassic of China [44], and the Early Cretaceous of Spain [45] have been recently removed from the clade [46]–[48].

Recent work has demonstrated the importance of heterodontosaurids for an understanding of ornithischian dinosaur evolution, particularly global patterns of ornithischian phylogeny, origination dates for major clades, and diversity patterns [7], [49], [50], as well as broader questions relating to early dinosaur evolution [51], [52] and the early evolution of feathers [30], [51], [53]. Although Ornithischia was one of the first fossil reptile groups to which cladistic approaches were applied [54]–[59], the study of global patterns of ornithischian interrelationships subsequently lagged behind that of the other major clades of dinosaurs, Theropoda and Sauropodomorpha. However, an increasing number of analyses of basal ornithischian phylogeny have been carried out in recent years [2], [7], [30], [41], [49], [50], [60], [61], with broad agreement on major ornithischian interrelationships. One key point on which these analyses disagree is the phylogenetic position of Heterodontosauridae. Sereno ([2], [56], [59]; see also [12], [13], [62]) has argued that heterodontosaurids represent the most basal grouping within Ornithopoda, a clade that also includes ‘hypsilophodontids’, iguanodontians, and hadrosaurs. By contrast, Butler *et al.* ([7], [50], [61]; see also [30], [41], [63]) have argued that heterodontosaurids are the most basal radiation of ornithischians, a position that appears to be more concordant with stratigraphic evidence, while several authors [13], [49], [54], [57], [64] have found support for a link between marginocephalians (Pachycephalosauria+Ceratopsia) and Heterodontosauridae. Finally, Heterodontosauridae has also been placed as a sister taxon to Cerapoda (Ornithopoda+Marginocephalia [58], [60]). The character evidence supporting these alternative placements was discussed critically by Norman *et al.* [14].

Inconsistencies regarding the phylogenetic position of heterodontosaurids are likely to be resolved with increased taxonomic sampling and a better understanding of the postcranial anatomy of these dinosaurs. The morphology of *Fruitadens haagarorum* [61] from the Late Jurassic Morrison Formation of the western USA fills an important gap in our knowledge of heterodontosaurid anatomy. Given its well-preserved postcranial material, the morphology of *Fruitadens* thus sheds new light into the monophyly, phylogenetic position, and evolutionary patterns of the clade. The preserved cranial material of *Fruitadens* also provides new information on heterodontosaurid functional morphology and craniodental variation. Furthermore, being the first heterodontosaurid for which published histological data are available, *Fruitadens* provides insights into the ontogeny and body size distribution of ornithischians. Because of the importance of this taxon for understanding heterodontosaurid evolution, we provide here a full description of its anatomy with detailed comparisons to other heterodontosaurids. Moreover, we provide initial analyses of the cranial functional morphology of late-surviving small-bodied heterodontosaurids, review characters supporting heterodontosaurid monophyly, and provide an overview of the evolutionary history of the group.

### Taxonomic background

The ornithischian *Echinodon becklesii* Owen, 1861 [65] is based upon fragmentary cranial material from the lowermost Cretaceous (Middle Purbeck Beds, Purbeck Limestone Group: Berriasian) of southern England, UK [32], [33], [37], [39], [65]. This material was originally described as ‘lacertilian’ (i.e. as a lizard) by Owen [65]; however, the dinosaurian nature of *Echinodon* was later noted by Owen ([66]: 9) (as ‘the small Purbeck Dinosaur [*Echinodon*]’) and Lydekker [67]. *Echinodon* has since been assigned to multiple phylogenetically disparate groups within Ornithischia, including Stegosauria, Thyreophora, “Hypsilophodontidae", and “Fabrosauridae" (see review in Norman & Barrett [37]). Most recently, *Echinodon* has been proposed to represent a Cretaceous heterodontosaurid [1], [13], [33]–[38] and Norman & Barrett [37] cited three potential synapomorphies supporting this referral: 1) a wedge-shaped predentary; 2) teeth from the midpoint of the maxillary/dentary tooth rows have denticles restricted to the apical-most third of their crowns; 3) absence of replacement foramina on the medial surface of the maxilla and dentary.

Callison & Quimby ([68]: figs 3B, C) figured a femoral shaft and a distal tibia with an articulated astragalus and calcaneum as those of a small “fabrosaurid" ornithischian dinosaur. These bones came from the Upper Jurassic Morrison Formation of the Fruita Paleontological Area (FPA), northwest of Grand Junction, Colorado, USA. The material was subsequently identified as *Echinodon* sp. on the basis of an initial assessment of the morphology of another specimen, consisting of associated jaws with teeth [34], [69]. Galton [36] proposed several autapomorphies for *Echinodon* based upon the English and Fruita material, such as the form of the dentary symphysis and the presence of an anteromedially directed edge on the distal part of the tibia. In addition, he listed several cranial and postcranial character synapomorphies shared by *Echinodon* (postcranial characters based on Fruita material) and *Heterodontosaurus*.

Galton [38] compared the morphology of the dentition of the Fruita material to *Echinodon*, and noted several differences. Subsequently, Butler *et al.* [61] erected the new taxon *Fruitadens haagarorum* for the Fruita material.

### Geological background

General accounts of the Fruita Paleontological Area (FPA – land administered by the Bureau of Land Management of the USA), including the history of discovery, geology, taphonomy and paleoenvironments, and fauna, are given by Callison [69], Kirkland [70], [71], and Foster [72]. Fossils at the FPA were collected from a geographically small area, covering approximately one square kilometer ([71]: fig. 4A). The sediments at the FPA are interpreted as representing a number of depositional environments, including low-sinuosity anastomosing river channels, levees, floodplains, and ponds [71]. Vertebrate fossils occur in nearly all of these facies, but the small-bodied vertebrate remains for which the FPA is famous occur in the so-called ‘drab floodplain’ and ‘alkaline pond’ facies, the former representing a poorly drained floodplain with poorly developed paleosols and the latter representing ephemeral floodplain ponds [71]. Preservation of these small vertebrate taxa is attributed to the alkaline nature of the enclosing sediments [71].

The vertebrate fauna documented from the FPA is diverse and includes dipnoan fish (*Ceratodus guentheri*), an amioid, the actinopterygian *Hulettia hawesi*, the chelonian *Glyptops*, the rhynchocephalians *Opisthias* and *Eilenodon robustus*, lizards including the anguimorphs *Parviraptor gilmorei* and *Dorsetisaurus* and the scincomorphs *Paramacellodus* and *Saurillodon*, a small cursorial mesosuchian crocodiliform, the sphenosuchian crocodiliform *Macelognathus vagans*, the mammals *Priacodon fruitaensis*, *Glirodon grandis* and *Fruitafossor windsheffeli*, the theropod dinosaurs *Ceratosaurus magnicornis* and *Allosaurus*, the sauropod dinosaurs *Camarasaurus* and *Apatosaurus*, and the large-bodied ornithischians *Stegosaurus* and *Dryosaurus* [71]–[85].

### Institutional abbreviations

NHMUK [formerly NHM, BMNH], Natural History Museum, London, UK; IVPP, Institute of Vertebrate Paleontology and Paleoanthropology, Bejing, People's Republic of China; LACM, Dinosaur Institute of the Natural History Museum of Los Angeles County, Los Angeles, California, USA; MNA, Museum of Northern Arizona, Flagstaff, Arizona, USA; SAM-PK, Iziko South African Museum, Cape Town, South Africa.

## Results

### Systematic Paleontology

Dinosauria Owen, 1842 [86]

Ornithischia Seeley, 1887 [87]

Heterodontosauridae Kuhn, 1966 [88]

#### Phylogenetic definition

The most inclusive clade containing *Heterodontosaurus tucki* Crompton & Charig, 1962 [10] but not *Parasaurolophus walkeri* Parks, 1922 [89], *Pachycephalosaurus wyomingensis* (Gilmore, 1931) [90], *Triceratops horridus* Marsh, 1889 [91], *Ankylosaurus magniventris* Brown, 1908 [92] (Sereno [93]).

#### Diagnosis

Small-bodied ornithischians diagnosed by the following unique combination of characters [61]: (1) three premaxillary teeth; (2) arched and recessed diastema between the premaxilla and the maxilla; (3) wedge-shaped predentary; (4) constriction on the proximal surface of the humerus, between the head and the medial tubercle; (5) ‘rod-like’ (with near parallel sides) fourth trochanter on the femur; (6) very slender distal fibula; (7) fused astragalus and calcaneum (astragalocalcaneum); (8) proximal phalanges of pedal digits II–IV with extensor pits on distal heads.

*Fruitadens haagarorum* Butler, Galton, Porro, Chiappe, Henderson & Erickson, 2010 [61]

Figs. 1, 2, 3, 4, 5, 6, 7, 8A, B, 9, 10, 11, 12, 13, 14, 15, 16

**Figure 1:**
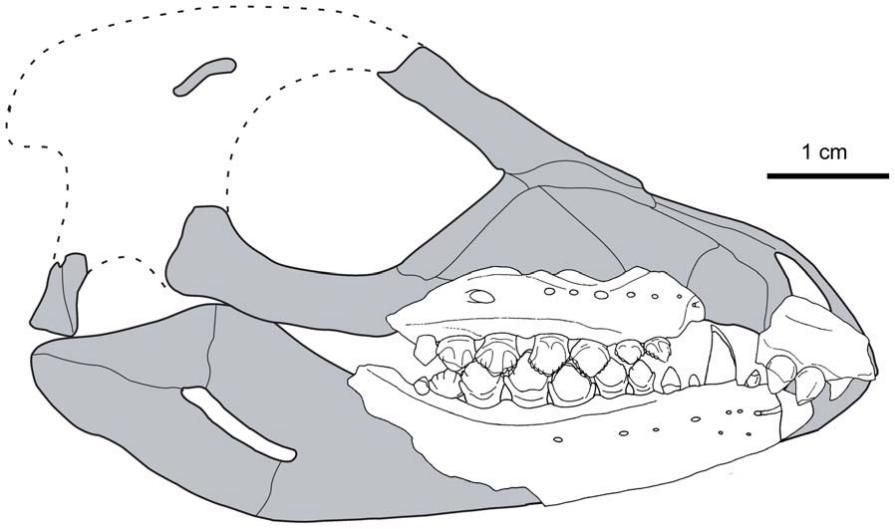
Cranial reconstruction of *Fruitadens haagororum*. Fragments representing cranial and mandibular material preserved in LACM 115747 and 128258 are superimposed on the preserved skull of *Tianyulong confuciusi* (shown in gray). Outline of posterior cranium, extrapolated from *Heterodontosaurus tucki*, shown by dotted lines.

**Figure 2:**
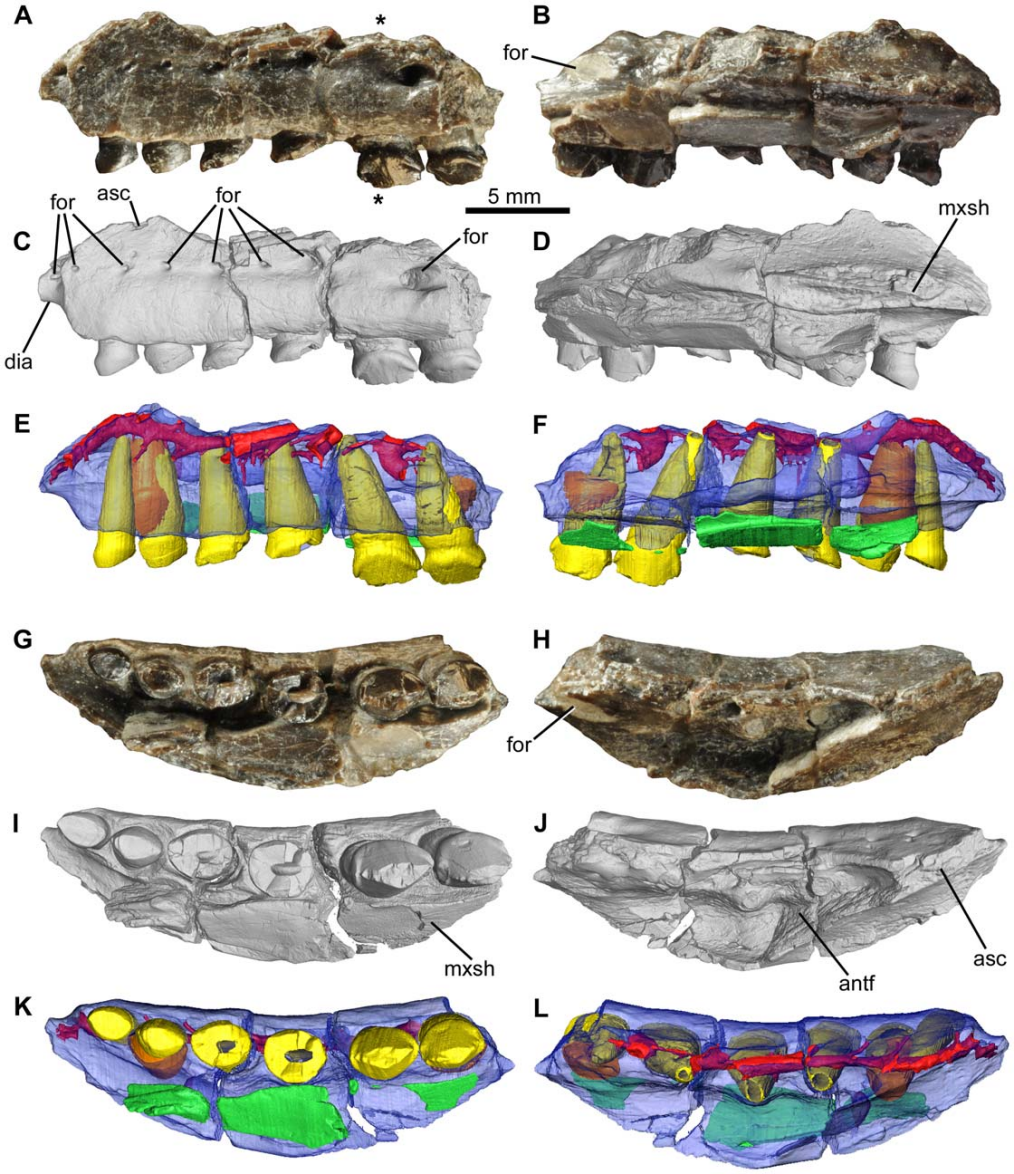
*Fruitadens haagarorum*, LACM 115747 (holotype), left maxilla. Maxilla in lateral (A, C, E), medial (B, D, F), ventral (G, I, K), and dorsal (H, J, L) views, with photographs (A, B, G, H), external renderings from μCT data (C, D, I, J), and reconstructions from μCT data (E, F, K, L). See also video S1. Elements in the CT reconstructions are colour-coded as follows: maxilla, blue; functional teeth, yellow; replacement teeth, orange; internal canals, red; palatal (vomer?) fragment, green. Asterisks mark the position of the transverse μCT cross section in Figure 3. Abbreviations: antf, antorbital fossa; asc, broken base of ascending process; dia, diastema between premaxilla and maxilla; for, foramen; mxsh, maxillary shelf.

**Figure 3:**
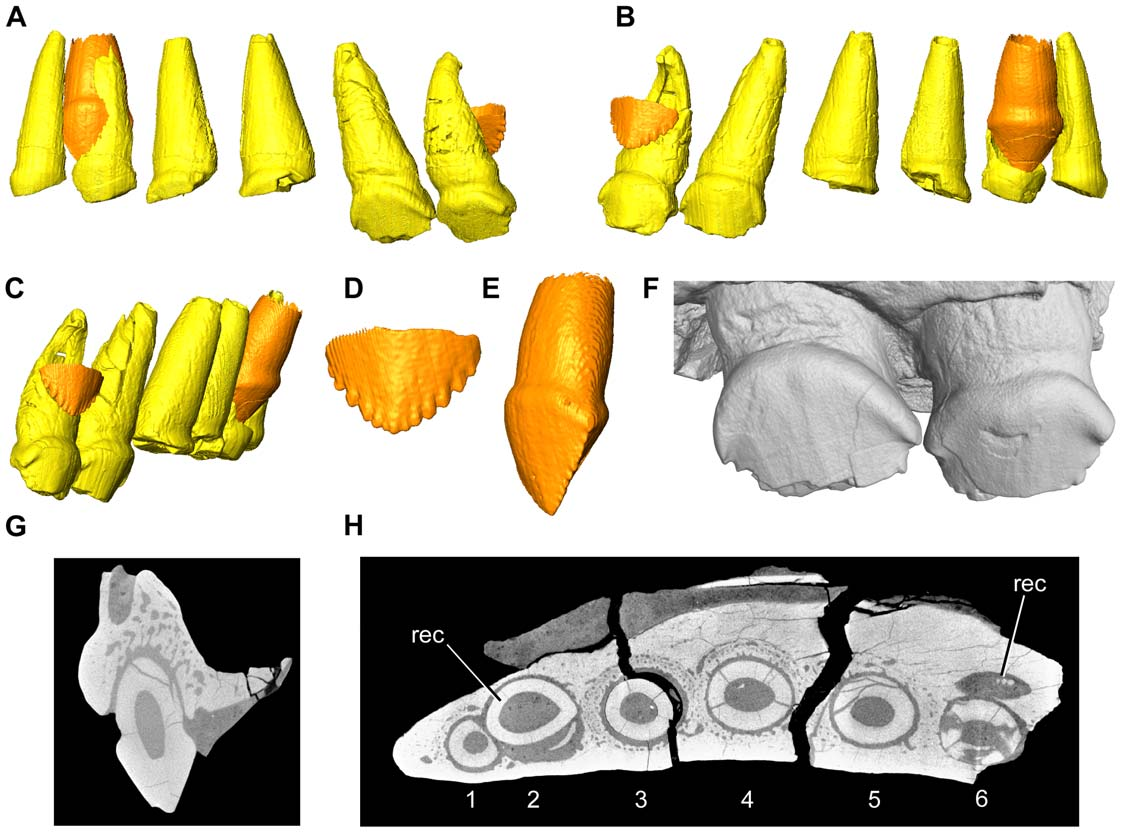
*Fruitadens haagarorum*, LACM 115747 (holotype), μCT data for the dentition of the left maxilla. Reconstructed dentition (surrounding bone removed) in lateral (A), medial (B), and posteromedial (C) views. Reconstructed posterior replacement tooth in medial view (D), and reconstructed anterior replacement tooth in anteromedial view (E). External renderings of crowns 5 and 6 in lateral view (F). Transverse (G) and longitudinal (H) μCT cross sections through maxilla (see asterisks in Figure 2A for the position of the transverse cross section) showing the spongy bone surrounding the tooth roots. Elements in the CT reconstructions are colour-coded as follows: functional teeth, yellow; replacement teeth, orange. Abbreviation: rec, replacement crown. Numbers indicate tooth positions from anterior to posterior.

**Figure 4:**
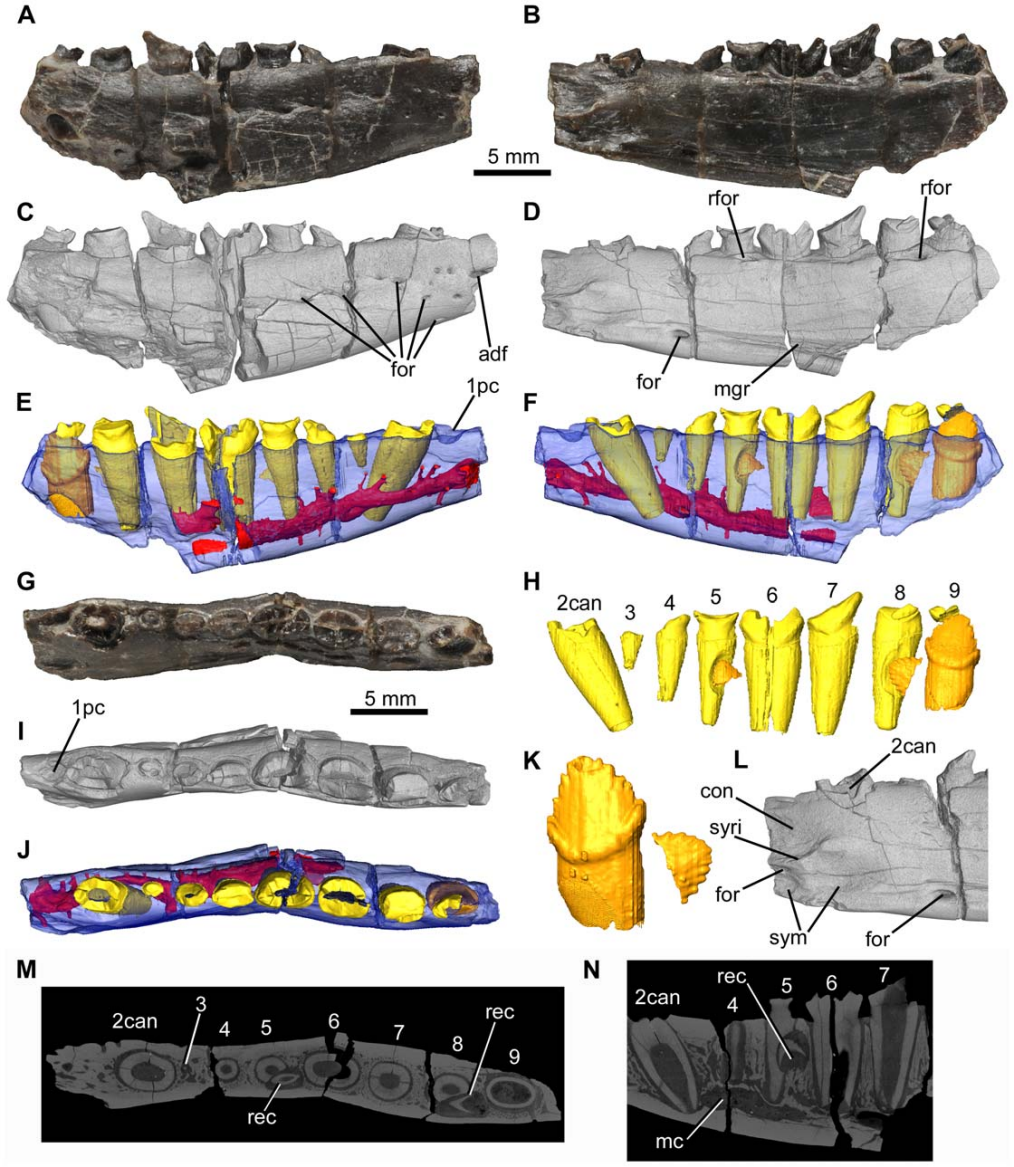
*Fruitadens haagarorum*, LACM 115747 (holotype), right dentary. Dentary in lateral (A, C, E), medial (B, D, F), and dorsal (G, I, J) views, with photographs (A, B, G), external renderings from μCT data (C, D, I), and reconstructions from μCT data (E, F, J). Reconstructed and extracted dentition in medial view (H). Close-up of the reconstructed and extracted posterior replacement teeth in lateral view (K). Close-up of the external rendering showing the symphyseal region in medial view (L). Longitudinal CT slice (M) through the entire element and sagittal CT slice (N) through the anterior part of the mandible. See also video S2. Elements in the CT reconstructions are colour-coded as follows: dentary, blue; functional teeth, yellow; replacement teeth, orange; internal canals, red. The dentary has been made transparent in order to better visualize its internal anatomy. Abbreviations: 1pc, position of the ‘pre-caniniform’ (missing in this specimen); 2can, caniniform tooth in second tooth position; adf, anterior dentary foramen; con, concavity dorsal to the symphyseal surface; for, foramina; mc, mandibular canal within the dentary; mgr, Meckelian groove; rec, replacement crown; rfor, replacement foramen; sym, symphyseal surface; syri, curved ridge marking dorsal margin of symphysis. Numbers indicate tooth positions from anterior to posterior.

**Figure 5:**
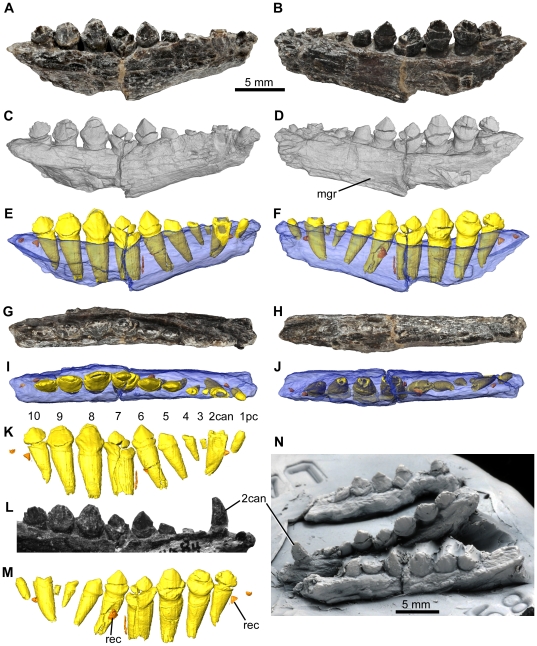
*Fruitadens haagarorum*, LACM 128258, right dentary. Dentary in lateral (A, C, E), medial (B, D, F), dorsal (G, I), and ventral (H, J) views, with photographs (A, B, G, H), external renderings from μCT data (C, D), and CT reconstructions from μCT data (E, F, I, J). Reconstructed and extracted dentition in lateral (K) and medial (M) views. Photograph of the dentary in lateral view by PMG in the 1980s before the caniniform was damaged and lost (L). Photograph of a cast of the maxilla and left and right dentaries of LACM 128258 as originally preserved (N). See also video S3. Elements in the CT reconstructions are colour-coded as follows: dentary, blue; functional teeth, yellow; replacement teeth, orange. The dentary has been made transparent in order to better visualize its internal anatomy. Abbreviations: 1pc, ‘pre-caniniform’; 2can, caniniform tooth in second tooth position; mgr, Meckelian groove; rec, replacement crown. Numbers indicate tooth positions from anterior to posterior.

**Figure 6:**
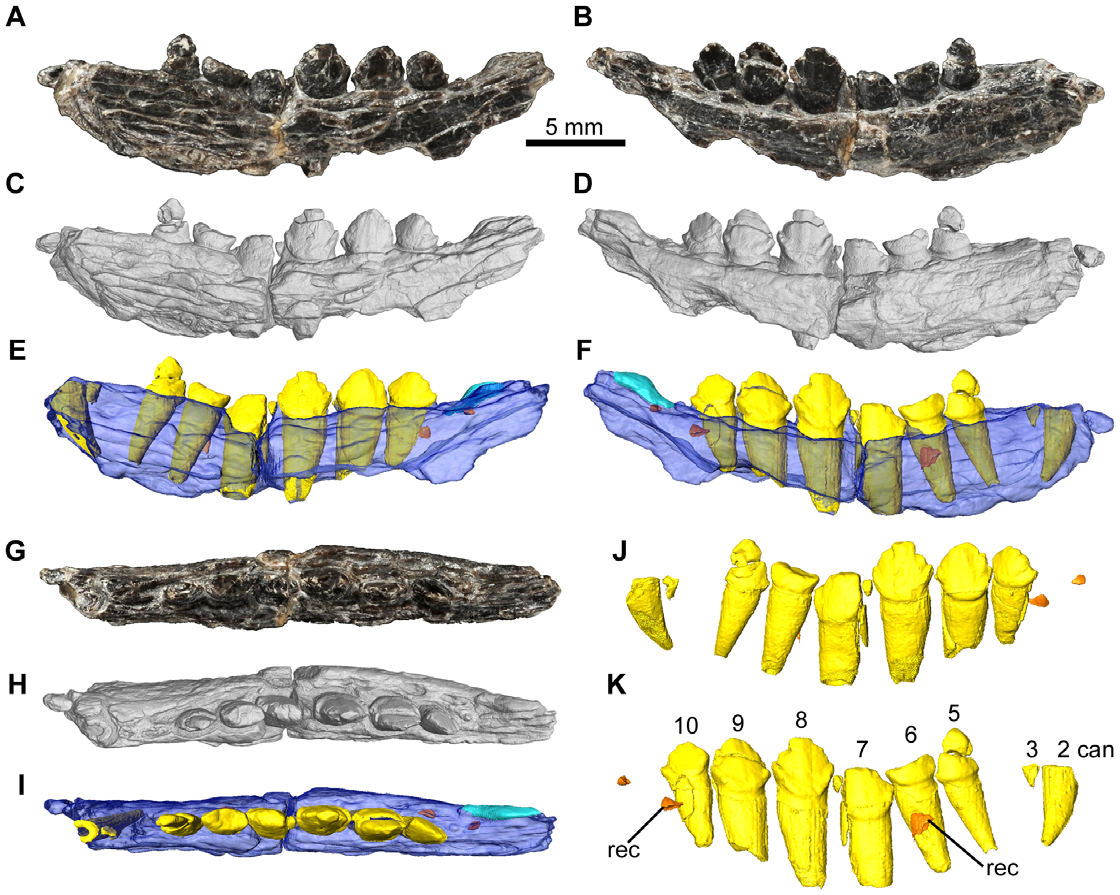
*Fruitadens haagarorum*, LACM 128258, left dentary. Dentary in lateral (A, C, E), medial (B, D, F), and dorsal (G, H, I) views, with photographs (A, B, G), external renderings from μCT data (C, D, H), and CT reconstructions from μCT data (E, F, I). Reconstructed and extracted dentition in lateral (J) and medial (K) views. Elements in the CT reconstructions are colour-coded as follows: dentary, dark blue; fragment of coronoid, light blue; functional teeth, yellow; replacement teeth, orange. The dentary has been made transparent in order to better visualize its internal anatomy. Abbreviations: 2can, caniniform tooth in second tooth position; rec, replacement crown. Numbers indicate tooth positions from anterior to posterior.

**Figure 7:**
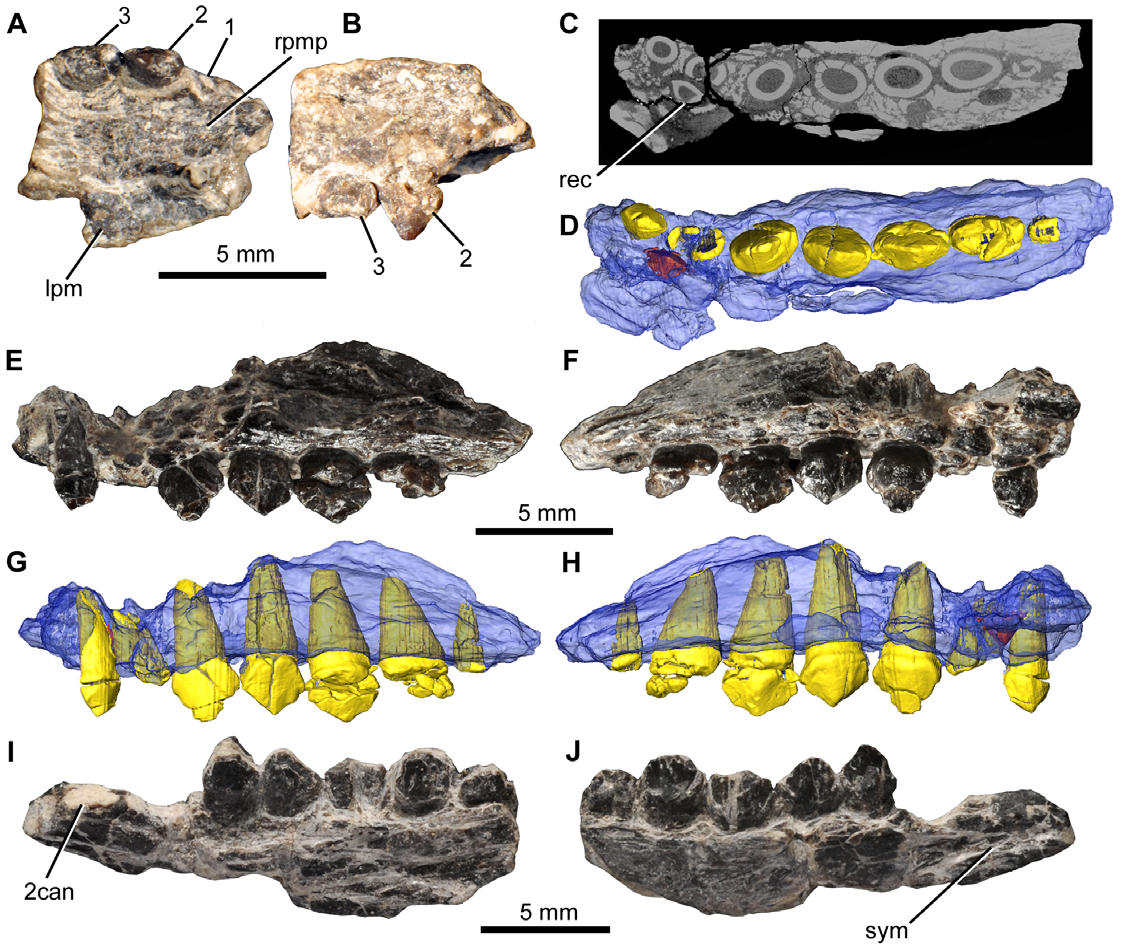
*Fruitadens haagarorum*, cranial bones. LACM 128258, right premaxilla in ventral (A) and lateral (B) views. LACM 128258, partial left maxilla, μCT slice (C), and ventral (D), lateral (E, G), and medial (F, H) views, as photographs (E, F) and reconstruction from μCT data (D, G, H). LACM 128303, partial left dentary in lateral (I) and medial (J) views. Elements in the CT reconstructions are colour-coded as follows: maxilla, blue; functional teeth, yellow; replacement teeth, orange. The maxilla has been made transparent in order to better visualize its internal anatomy. Abbreviations: 2can, position of the caniniform tooth (missing); lpm, left premaxilla; rc, replacement crown; rpmp, right premaxillary palate; sym, dentary symphysis. Numbers indicate tooth positions from anterior to posterior.

**Figure 8:**
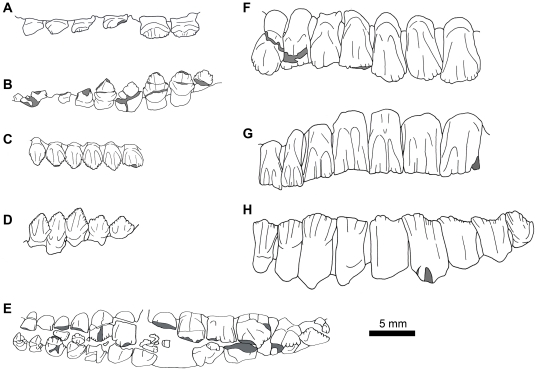
The post-caniniform (“cheek") tooth rows of various heterodontosaurid genera. Anterior end of the tooth row is always to the left (some images have been flipped horizontally). Areas of breakage are shaded in gray. A. *Fruitadens haagarorum* (holotype, LACM 115747), left maxillary teeth in labial view. B. *Fruitadens haagarorum* (LACM 128258), right dentary teeth in lingual view. C. *Echinodon becklesii* (NHMUK OR 48211), right maxilla in labial view (image has been flipped horizontally). D. *Echinodon becklesii* (NHMUK OR 48213), left dentary in lingual view (image has been flipped horizontally). E. *Abrictosaurus consors* (NHMUK RU B54), left maxillary and dentary (partially obscured) tooth rows in labial view. F. *“Lanasaurus scalpridens"* (BPI 4244), left maxillary teeth 2–8 in labial view. G. *Heterodontosaurus tucki* (SAM-PK-K1332), left maxillary teeth 2–8 in labial view. H. *Heterodontosaurus tucki* (SAM-PK-K1332), right dentary teeth 3–11 in lingual view.

**Figure 9:**
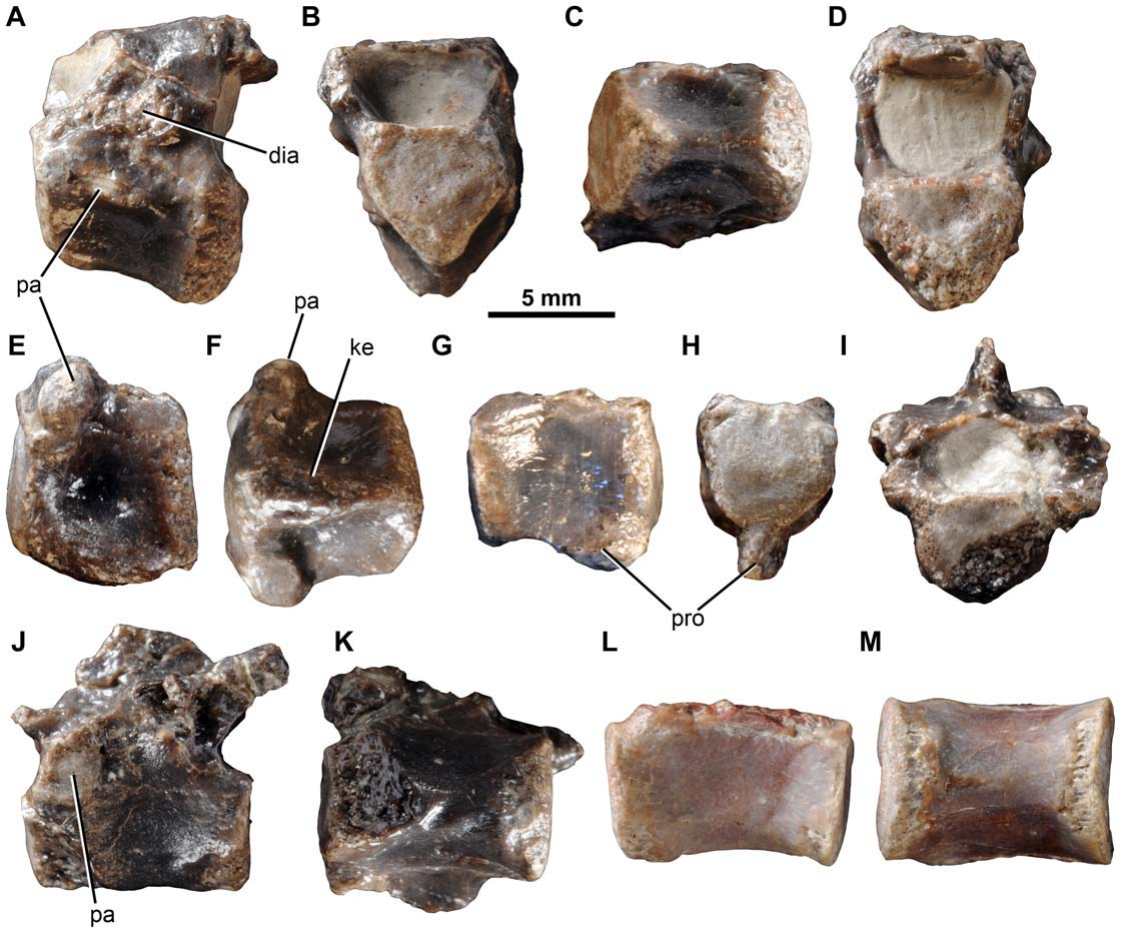
*Fruitadens haagarorum*, LACM 115747 (holotype), cervical and dorsal vertebrae. Anterior cervical vertebra in left lateral (A), anterior (B), ventral (C) and posterior (D) views. Posterior cervical centrum in left lateral (E) and ventral (F) views. Anterior dorsal centrum in right lateral (G) and anterior (H) views. Dorsal vertebra in anterior (I), lateral (J) and ventral (K) views. Posterior dorsal centrum in lateral (L) and ventral (M) views. Abbreviations: dia, diapophysis; ke, keel; pa, parapophysis; pro, ventral projection.

**Figure 10:**
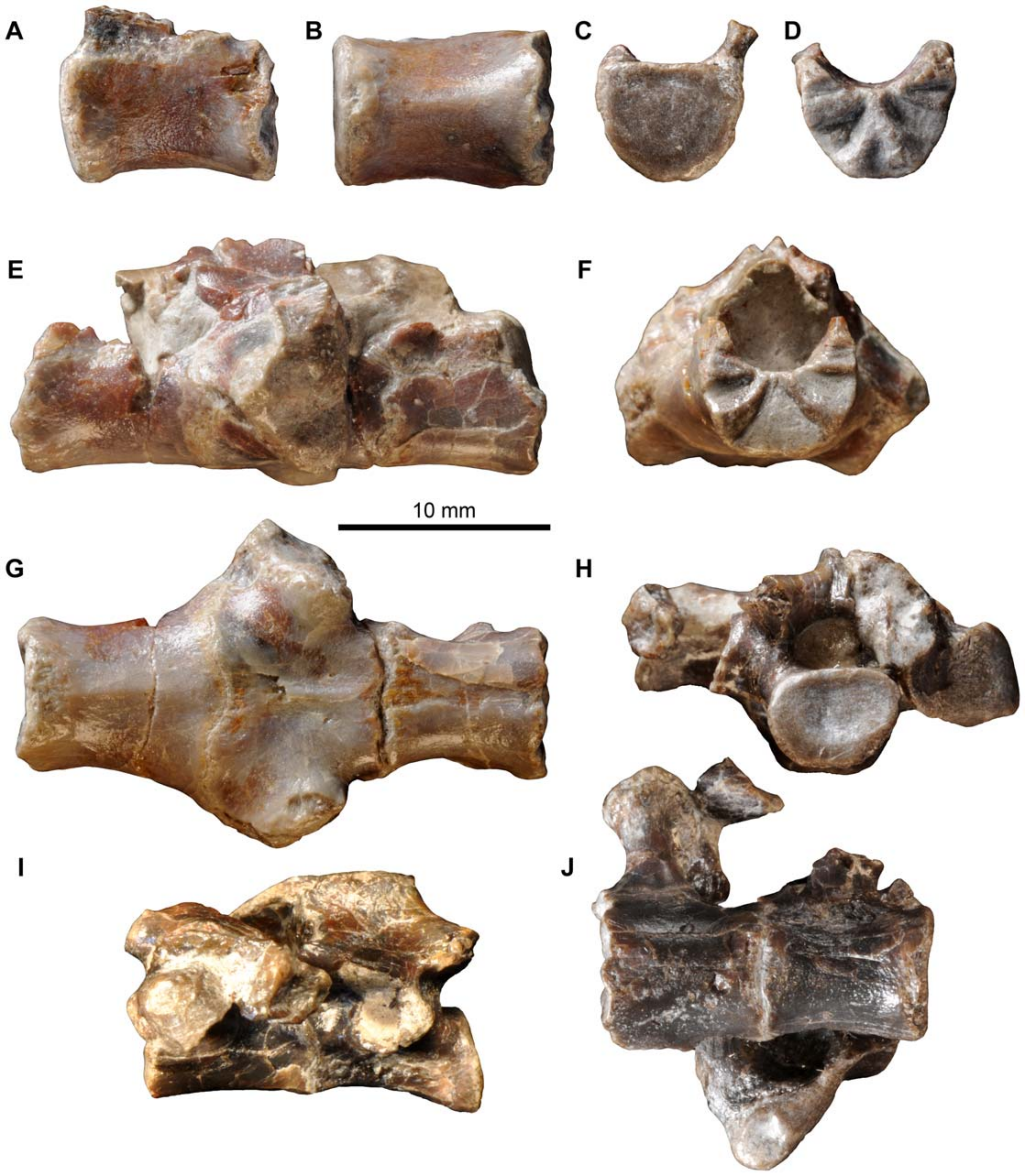
*Fruitadens haagarorum*, LACM 115747 (holotype), sacral vertebrae. First sacral vertebra in left lateral (A), ventral (B), anterior (C), and posterior (D) views. Sacral vertebrae 2–4 in left lateral (E), anterior (F), and ventral (G) views. Sacral vertebrae 5 and 6 in posterior (H), left lateral (I) and ventral (J) views.

**Figure 11:**
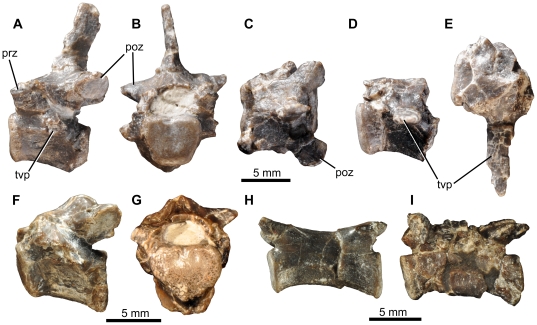
*Fruitadens haagarorum*, LACM 115747 (holotype), caudal vertebrae. Anterior caudal vertebrae in left lateral (A, D, F), anterior (B, G), and dorsal (C, E) views. Distal caudal vertebrae in left lateral view (H, I). Abbreviations: poz, postzygapophysis; prz, prezygapophysis; tvp, transverse process.

**Figure 12:**
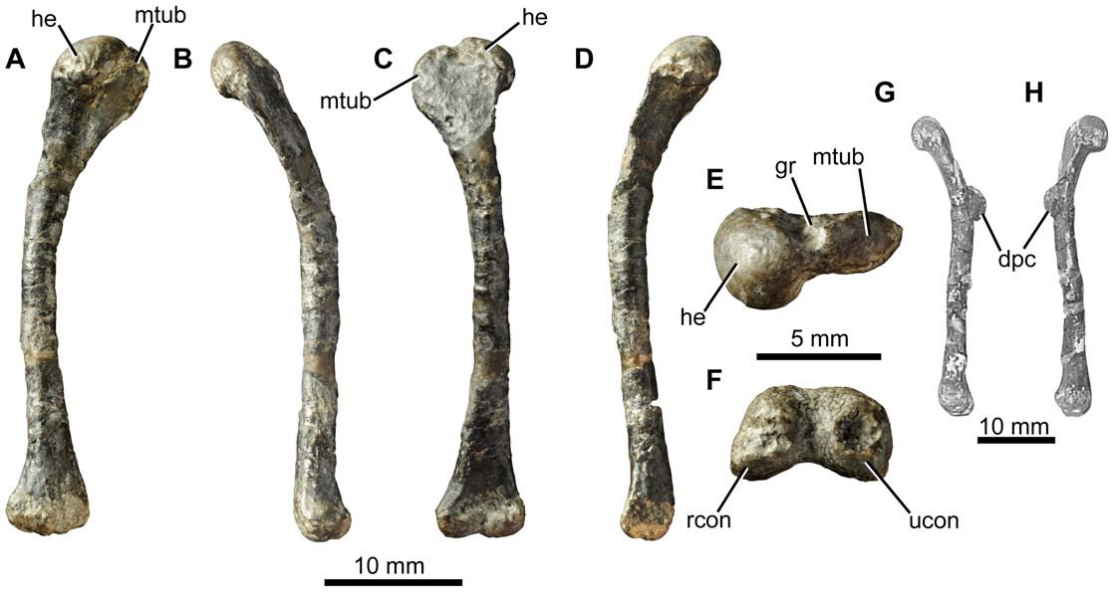
*Fruitadens haagarorum*, LACM 120478, left humerus. Humerus in posterior (A) medial (B), anterior (C), lateral (D), proximal (E) and distal (F) views. Left humerus as originally preserved, prior to damage to the deltopectoral crest (photographs taken by PMG). Abbreviations: dpc, deltopectoral crest; gr, groove on proximal surface; he, head; mtub, medial tubercle; rcon, radial condyle; ucon, ulnar condyle.

**Figure 13:**
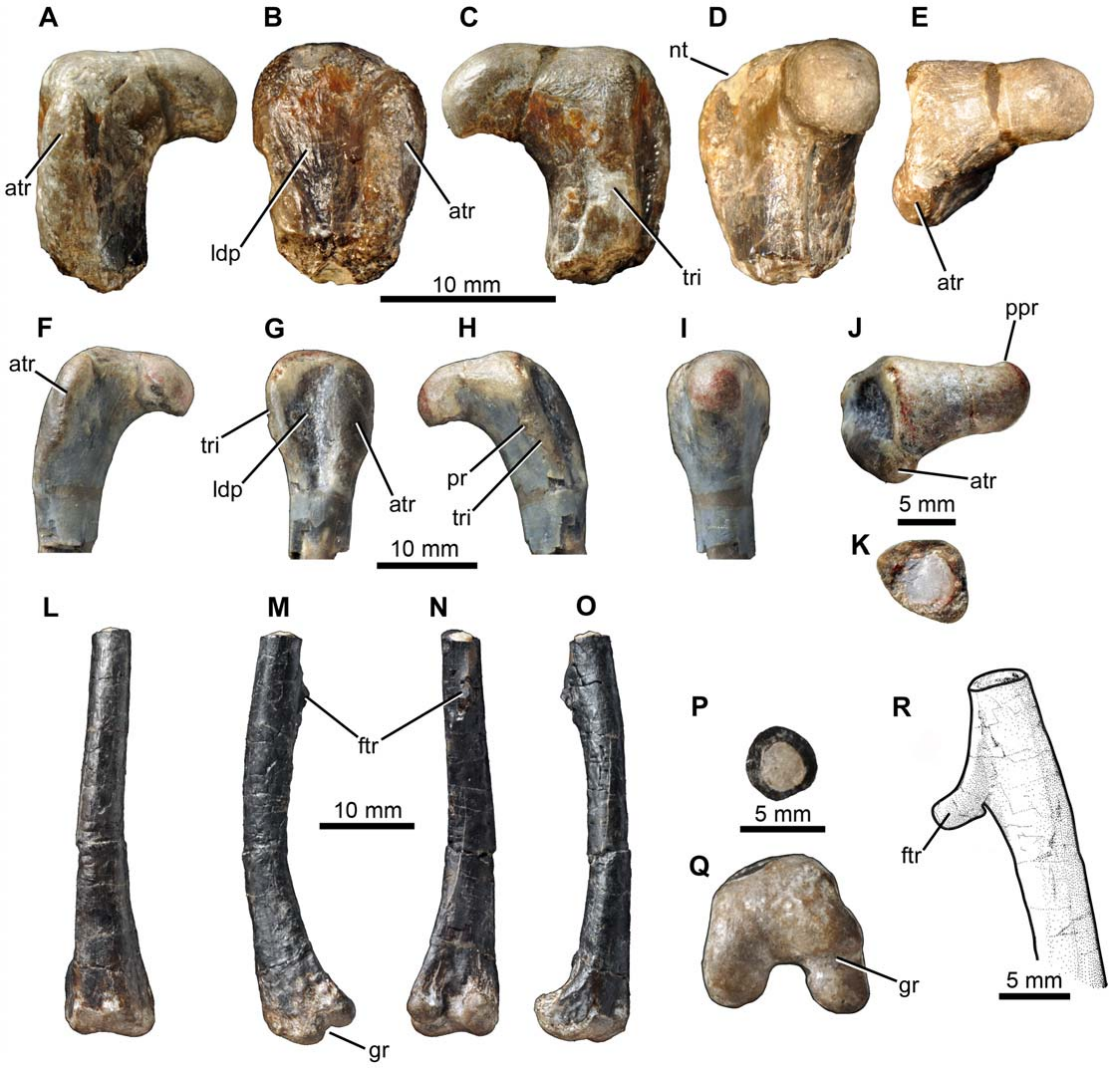
*Fruitadens haagarorum*, femoral anatomy. LACM 115747 (holotype), proximal right femur in anterior (A), lateral (B), posterior (C), medial (D) and proximal (E) views. LACM 115727, proximal right femur in anterior (F), lateral (G), posterior (H), medial (I) and proximal (J) views. Note that only the proximal portion of the shaft is shown; more distal parts are preserved, but it is not clear if they have been reconstructed correctly. LACM 115727, cross section through the femur at approximately mid-length (K). LACM 120478, distal left femur in anterior (L), lateral (M), posterior (N), medial (O), cross-sectional (at preserved proximal end: P) and distal (Q) views. LACM 120478, sketch of the midshaft of the left femur as preserved in the 1980s, prior to damage to the fourth trochanter (modified from Callison & Quimby 1984). Abbreviations: atr, anterior trochanter; ftr, fourth trochanter; gr, transverse groove on distal femur; lpd, broad depression on lateral surface of greater trochanter; nt, shallow notch separating proximally the greater and anterior trochanters; ppr, subtle posterior projection of femoral head; pr, small knob-like projection on posterior surface of proximal end; tri, thickened ridge delimiting the posterior margin of the greater trochanter.

**Figure 14:**
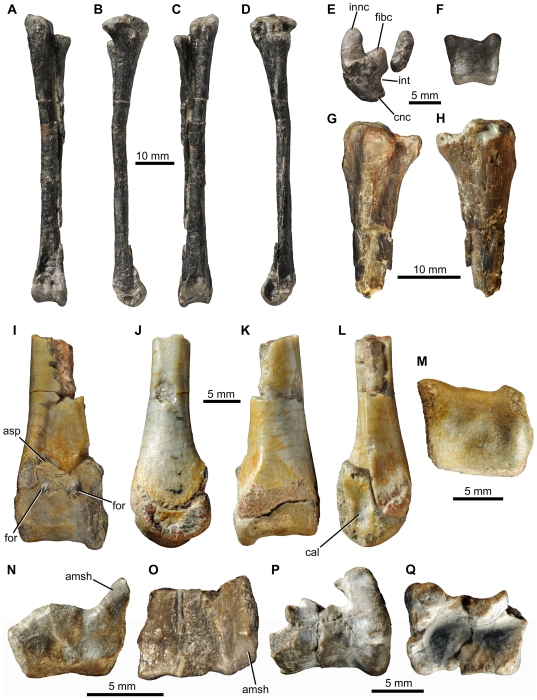
*Fruitadens haagarorum*, distal hindlimb. LACM 120478, articulated left tibia, fibula and astragalocalcaneum in anterior (A), medial (B), posterior (C), lateral (D), proximal (E) and distal (F) views. LACM 115747 (holotype), proximal left tibia in lateral (G) and medial (H) views. LACM 115727, distal left tibia with attached astragalocalcaneum in anterior (I), medial (J), posterior (K), lateral (L) and distal (M) views. LACM 115747 (holotype), distal right tibia in distal (N) and anterior (O) views. LACM 120602, left astragalocalcaneum in anterior (P) and proximal (Q) views. Abbreviations: amsh, anteromedial sheet of tibia; asp, ascending process; cal, calcaneum; cnc, cnemial crest; fibc, fibular condyle; for, foramen; innc, inner condyle; int, notch between inner condyle and fibular condyle.

**Figure 15:**
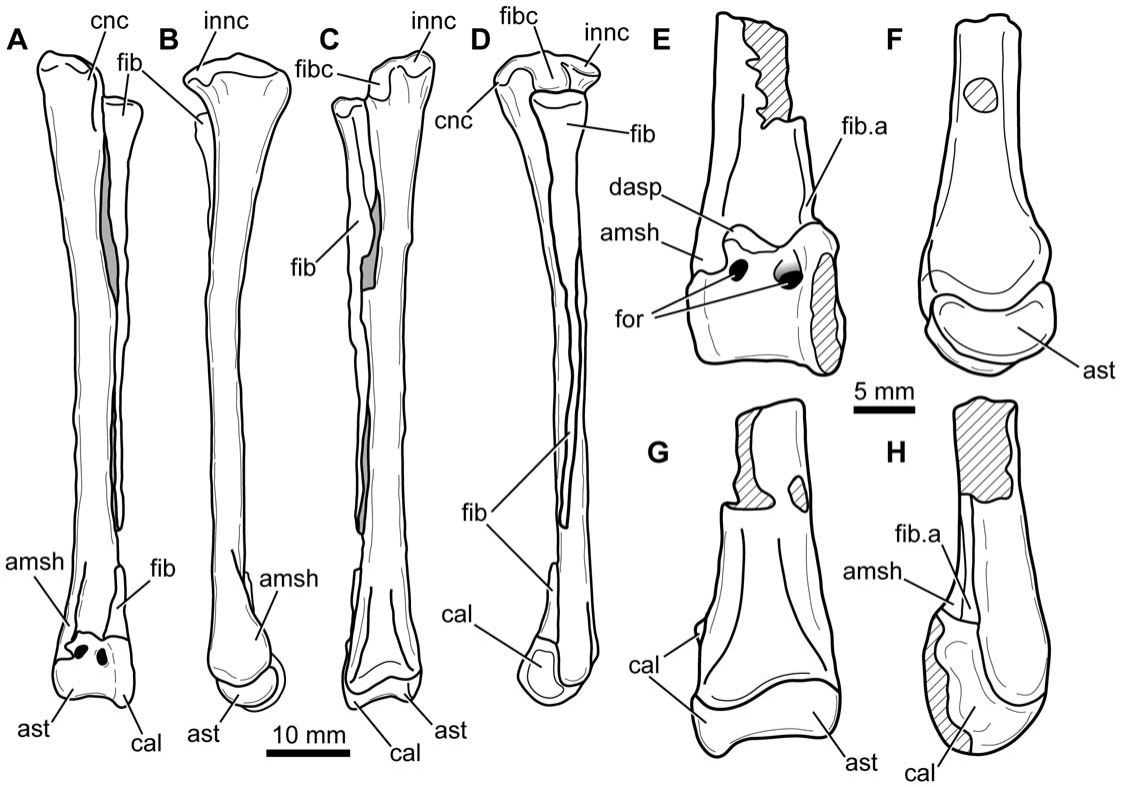
*Fruitadens haagarorum*, line drawings of distal hindlimb. LACM 120478, articulated left tibia, fibula and astragalocalcaneum in anterior (A), medial (B), posterior (C), and lateral (D) views. LACM 115727, distal left tibia with attached astragalocalcaneum in anterior (E), medial (F), posterior (G), and lateral (H) views. Abbreviations: amsh, anteromedial sheet of tibia; ast, astragalus; cal, calcaneum; cnc, cnemial crest; dasp, dorsal part of ascending process, formed by separate ossification; fib, fibula; fib.a, articular surface for fibula; fibc, fibular condyle; for, foramen; innc, inner condyle.

**Figure 16:**
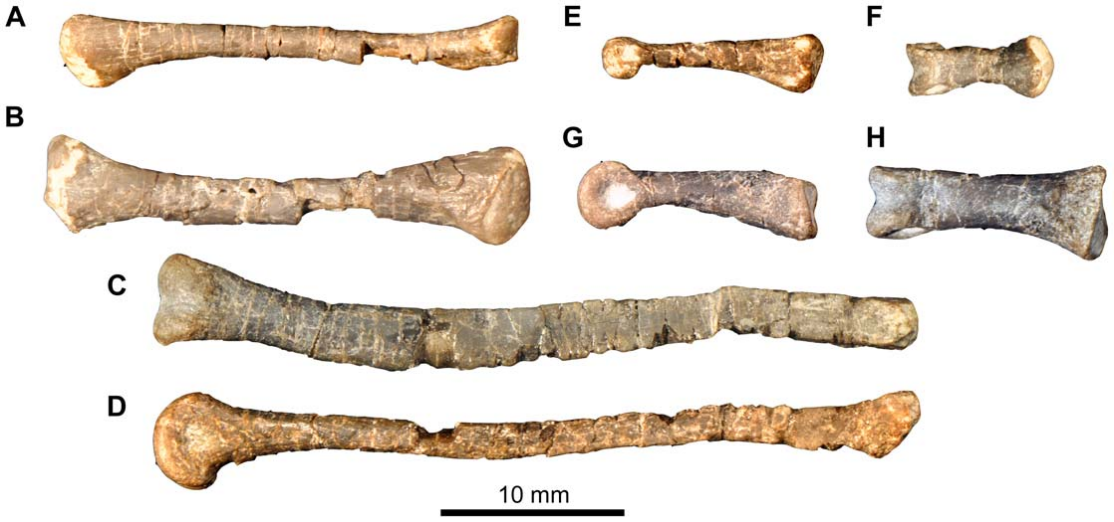
*Fruitadens haagarorum*, LACM 120602, metatarsals and pedal phalanges. Left metatarsal I in dorsal view (A). Right metatarsal I in lateral view (B). Additional metatarsal (position uncertain) in dorsal (C) and medial or lateral (D) views. Phalanx I-1 in lateral or medial view (E). Phalanx (position uncertain) in dorsal view (F). Phalanx III-1 in lateral or medial (G) and dorsal (H) views.

“cf. *Coelurosaurus*"; Callison & Rasmussen, 1980:151 [94]

“cf. *Fabrosaurus*"; Callison & Rasmussen, 1980:153 [94]

“Fruita fabrosaurid"; Callison & Quimby, 1984:figs 3B, C [68]

“*Echinodon* sp."; Callison, 1987:95, fig. 4 [69]

“Gen. & sp. nov., Morrison Formation"; Olshevsky & Ford, 1994:93, fig. 43 [34]

“*Echinodon* sp."; Galton, 2002:55–56A [36]

“Fruita jaws"; Galton, 2006:26, 28, fig. 2.7A–G [38]

#### Diagnosis

Small heterodontosaurid ornithischian characterised by the following unique combination of characters, including autapomorphies (* indicates character that is autapomorphic within Heterodontosauridae; ** indicates character that is autapomorphic within Ornithischia): (1) premaxillary crowns small and subequal to one another in size, expanded labiolingually and mesiodistally above the root; (2) maxillary caniniform absent; (3) maxillary and dentary crowns apicobasally low and triangular in lingual and labial views, with symmetrically distributed enamel; (4*) mesial and distal denticles extend over half of the apicobasal height of maxillary and dentary crowns, not restricted to apical third; (5*) dentary caniniform present but erupted apicobasal height does not exceed that of the crown of the largest dentary ‘cheek’ (post-caniniform) tooth; (6**) small, unserrated, peg-like and procumbent tooth present anterior to dentary caniniform; (7**) small foramen on anteroventral aspect of the medial dentary, ventral to the Meckelian groove and beneath dentary crowns 3 and 4; (8) distal end of tibia with anteromedial flange; (9**) apex of the ascending process of astragalus is formed by a separate ossification; (10**) two large foramina pierce anterior surface of ascending process of astragalus (modified from Butler *et al.* [61]).

#### Etymology

*Fruitadens*, from Fruita (hypodigm locality) and *dens* (Latin, tooth); *haagarorum*, for Paul Haaga, Jr, Heather Haaga, Blythe Haaga, Paul Haaga III, and Catalina Haaga, to honour their support of the Natural History Museum of Los Angeles County (LACM, Los Angeles, USA), where the specimens of *Fruitadens haagarorum* are held.

#### Holotype

LACM 115747, associated jaws, vertebrae and limb bones of a near full-grown individual (Butler *et al.* [61]: figs 2B, E, I, 3A–C; cf. *Fabrosaurus* of Callison & Rasmussen [94]: 153). Includes maxillae (both incomplete), partial right dentary, and anterior end of left dentary, disarticulated vertebrae including two partial cervicals, six partial dorsals, six sacrals and numerous caudals, proximal end of the right femur, proximal and distal ends of both tibiae, partial metatarsal. Collected by J. M. Clark, August 1977, at “Locality Number 4" ([94]: 153), Fruita Paleontological Area (FPA). The specimen is currently catalogued as from locality LACM 4684: LACM 4684 is a “general locality for specimens from FPA with poor specific locality data" ([70]: 95).

#### Referred specimens

LACM 115727, proximal ends of both femora, proximal and distal ends of left tibia with attached astragalocalcaneum, bone fragments ([61]: figs 2G, H, 3F; [67]: fig. 3C) (referred to as cf. *Coelurosaurus* by Callison & Rasmussen ([94]: 151). Collected by G. L. Callison and party (July/August 1979). Callison & Rasmussen ([94]: 151) give the locality as “Locality Number 4" but it is currently catalogued as collected from locality LACM 5576, “George's ‘Coelurosaur’ Site" ([71]: 93).

LACM 120478, left humerus, partial left femur, and articulated left tibia, fibula, and astragalocalcaneum ([61]: fig. 2J–P; [68]: fig. 3B) (referred to as cf. *Coelurosaurus* by Callison & Rasmussen [94]: 151). Collected July/August 1979 by G. L. Callison and party, also at “Locality Number 4" ([94]: 151) and now catalogued as collected from LACM 5572, the “Main Callison Quarry" ([71]: 94).

LACM 120602, distal caudal vertebra, left astragalocalcaneum and elements of the metatarsus and pes. Collected 10^th^ June 1985 by G. L. Callison and party from locality LACM 4684 (see above).

LACM 128258, right premaxilla, partial left maxilla, originally articulated dentaries, dorsal vertebra, distal caudal vertebra ([34]: 79, fig. 14; [38]: figs 2.7A, B; [61]: figs 2A, C, D, F, 3D). A cast of the jaws of the dentaries (including the anteriorly positioned caniniform, which is no longer preserved in the original specimen) is held at the LACM. Collected by G. L. Callison and party (no date given) from locality LACM 4684 (see above).

LACM 128303, poorly preserved anterior left dentary (containing five crowns and four empty alveoli). Collected in 1981 by G. L. Callison and party from locality LACM 4684 (see above).

#### Horizon and type locality

All specimens came from the Morrison Formation at the LACM Fruita Paleontological Area (FPA), west of Fruita, 19 km northwest of Grand Junction, Mesa County, Colorado, USA ([61]: fig. 1.) The approximate latitude and longitude of the FPA is 39.2°N, 108.8°W. Specimens were collected in the late 1970s and early 1980s from the ‘drab flood-plain facies’ at the base of the Brushy Basin Member of the Morrison Formation immediately above the ‘clay change’ horizon ([61]: fig. S1; [71]). The ‘clay change’ horizon is commonly used for regional correlation of the Morrison Formation [95]. Turner & Peterson ([95]: fig. 7) placed the localities (listed as CO-33 in their stratigraphic sections and their Appendix 3) yielding *Fruitadens* within the Kimmeridgian, and within their ‘Dinosaur Zone 2’ and charophyte-ostracode Zone 4. Stratigraphic horizons closely equivalent to the Fruita quarries yield ^40^Ar/^39^Ar isotopic dates of 150.3±0.3 Ma and 150.2±0.5 Ma [95], [96]. This would suggest an early Tithonian age for the Fruita quarries based upon the most recent geological time scales [97], [98] that place the Tithonian at 150.8–145.5 Ma.

#### Notes on associations of specimens

No data on the original field associations of the holotype and referred specimens is currently available at the LACM. Within the holotype, all of the preserved material is generally consistent (in terms of size, morphology, lack of duplication of elements, and preservation) with belonging to a single individual. A distal femur of the crocodylomorph *Macelognathus* was previously included within LACM 115747, but has now been removed from this specimen. Each of the other specimens referred to *Fruitadens* likely represents a single individual, based upon consistent morphology and lack of overlapping elements.

### Description and comparisons

#### Skull anatomy: general comments

The skull is represented in the holotype by fragmentary maxillae (with only a few crowns preserved), most of the right dentary, and the anterior end of the left dentary. Most of the dentary crowns are missing, with only crown bases and/or just tooth roots preserved. In LACM 128258, the incomplete right premaxilla, left maxilla and dentaries are preserved, and most of the crowns are preserved (although damaged). LACM 128303 is a poorly preserved anterior left dentary. Other referred specimens lack cranial elements, and the morphology of the remainder of the skull is unknown. A tentative skull reconstruction is presented here (Fig. 1; modified from Butler *et al.* [61]), prepared by overlaying known skull elements from *Fruitadens* on an outline of the more complete skull of *Tianyulong* [30]), which more closely resembles *Fruitadens* in mandibular and dental morphology than do Early Jurassic heterodontosaurids (see below).

Measurements of the skull and postcranial skeleton are provided as a supplementary data file (Text S1).

#### Premaxilla

The right premaxilla (LACM 128258) is incomplete and the bone surface is poorly preserved (Fig. 7A, B). Part of the lateral surface and the bony palate are preserved, but the element is broken both at the anterior end and immediately posterior to the last tooth (which appears, however, to have been the final premaxillary tooth), so it is not possible to determine any contribution of the premaxilla to the diastema between the premaxilla and the maxilla (see below) or the nature of the articulation between the premaxilla and the maxilla. The preserved portion of the palate apparently reaches to the median suture, which may be marked by a thin line of sediment suggesting that a fragment of the anteromedial part of the bony palate of the left premaxilla is attached (Fig. 7A: lpm). The palate is very gently arched dorsally, rather than completely horizontal, and it becomes slightly wider transversely towards its posterior end. The lateral surface of the element is poorly preserved, but it is gently dorsoventrally convex immediately above the crowns: dorsal to this the lateral surface is damaged and the presence or absence of a subnarial fossa cannot be determined. Two tooth crowns are positioned adjacent to one another at the posterior end of the right premaxilla (Fig. 7A, B). Anterior to these there is a subcircular cross section through the root of a third tooth. No evidence exists for additional crowns anterior to this root (this region appears to be edentulous) and it is highly likely that the complete premaxillary tooth count was three (contra Galton [38] who suggested a count of five).

The premaxillary crowns are poorly preserved, with much of the labial surface of the second crown missing and the crown of the third tooth missing its apex. The second tooth has a crown with a subtriangular, weakly recurved outline in lateral view, and the crowns of both appear to be slightly expanded mesiodistally and transversely above the root. Several coarse denticles (poorly preserved) occur along the distal margin of the crown of the second tooth, although its mesial margin is too poorly preserved for the presence or absence of denticulation to be determined. The presence or absence of denticulation cannot be assessed for the crown of the third tooth. The crowns are transversely compressed, with a mesiodistal length that exceeds their transverse width. Labiolingual asymmetry in crown morphology cannot be adequately assessed due to preservation. No evidence exists for ornamentation (e.g. apicobasally extending ridges) on the labial or lingual surfaces. The transverse and mesiodistal widths of the crowns of the second and third teeth are very similar to one another; therefore the third crown does not appear to be enlarged. The first tooth may have had a slightly smaller crown, judging from the cross-section through its root, but this cannot be confirmed because it is broken. Overall, the premaxillary crowns appear to be relatively small without a progressive increase in size posteriorly.

A count of three premaxillary teeth also occurs in some other heterodontosaurids (e.g. *Heterodontosaurus*, SAM-PK-K337, SAM-PK-K1332; NHMUK RU A100, specimen referred to *Lycorhinus* by Thulborn [17] and Gow [25]). Although the premaxilla is poorly preserved, a count of three also apparently occurs in *Echinodon* [32], [39], [64]. Thulborn [19] reported only two premaxillary teeth in NHMUK RU B54, the holotype specimen of *Abrictosaurus*; however, a cross section through the root of a third tooth is visible ([37]; RJB pers. obs.). Only a single premaxillary tooth was reported in *Tianyulong* ([30]: fig. 1E). The premaxilla of *Manidens* is unknown [41]. Three premaxillary teeth also occur in pachycephalosaurs [99] and some, but not all, basal ceratopsians [49], [100]–[102]. By contrast, 5–6 premaxillary teeth occur in many other early ornithischians, including *Lesothosaurus* [33], early thyreophorans [103]–[104], *Agilisaurus* [105], and basal ornithopods [106]–[108]. Four or more premaxillary teeth typically occur in ornithischian outgroups [109], [110].

The morphology of the premaxillary crowns in *Fruitadens* differs from all other heterodontosaurids with the apparent exception of *Echinodon* [39], although this morphology may represent a retained plesiomorphy at the level of Ornithischia (due to the similarities with premaxillary crowns of other basal ornithischians). In *Heterodontosaurus* the premaxillary dentition consists of three crowns that increase in size posteriorly (SAM-PK-K337, K1332). The anterior two crowns are comparatively small, conical, recurved, lack serrations and are not markedly expanded above their roots. The caniniform third crown is greatly enlarged, recurved, with serrations along the distal surface, and is unexpanded above its root. This condition differs substantially from that of *Fruitadens* and *Echinodon*, in which the premaxillary crowns do not increase in size posteriorly, are expanded above their roots, and a caniniform is absent. A similar premaxillary dentition to that of *Heterodontosaurus* occurs in NHMUK RU A100 ([17]: fig. 2), although the caniniform third crown is not as enlarged (relative to the more anterior crowns) as in *Heterodontosaurus*. In *Abrictosaurus* (NHMUK RU B54; [19]: fig. 2) the crowns are conical and unexpanded above their roots, and increase in size posteriorly, although the last crown is relatively small compared to the caniniform teeth of *Heterodontosaurus* and NHMUK RU A100. The single, posteriorly positioned, premaxillary tooth of *Tianyulong* is enlarged, caniniform and not expanded above the root ([30]: fig. 1E).

The premaxillary crowns of *Fruitadens* and *Echinodon* appear to be most similar to those of basal ornithischians such as *Lesothosaurus* [33] and *Scutellosaurus* [103], which are also expanded labiolingually and mesiodistally above their roots and do not increase in size posteriorly. Similar premaxillary teeth occur in some basal ornithopods [106]–[108], ceratopsians (e.g. *Liaoceratops*, IVPP V12738) and pachycephalosaurs [111].

#### Maxilla

The holotype includes an incomplete left maxilla, containing six tooth positions (Figs 2, 3, supplementary video; identified as the posterior right maxilla by Galton [38]: fig. 2.7A). A fragment containing two teeth represents the anterior end of the right maxilla; a second fragment contains three crowns that represent right maxillary teeth 4–6 (based upon the large foramen on the posterolateral surface of this fragment). A small section of the right maxilla separating these fragments is therefore missing. The fragments from the right maxilla are missing most of their medial surfaces (including most of the medial part of the antorbital fossa) and do not add anatomical information that is not evident in the left maxilla; they will therefore not be described in detail. Additionally, there are two small fragments in the holotype that could be from either the maxillary or dentary tooth rows: one of them has a single partial crown and the second has two partial crowns (one of which is very small). The complete tooth count for the maxilla of the holotype is unknown (although a reconstruction suggests that a count of 7–8 is plausible: Fig. 1), as is the nature of the midline contact (if one occurred) between the maxillae.

The left maxilla of the holotype is broken anteriorly, posteriorly and dorsally (Fig. 2). In ventral view, the maxillary tooth row is arched inwards along its length, such that the lateral surface of the element is concave anteroposteriorly (Fig. 2G, I, K). The teeth are set laterally (with no buccal emargination) at the anterior end; posteriorly they are inset a short distance (equivalent to approximately half of their transverse width). The buccal emargination is therefore very weakly developed in *Fruitadens*.

Dorsal to the crowns and ventral to the line of nutrient foramina (see below), the lateral surface of the maxilla is dorsoventrally convex; this convexity becomes more pronounced posteriorly, forming a low rounded shelf dorsal to the weak buccal emargination. Immediately beneath the broken base of the ascending process of the maxilla and the ventral margin of the external antorbital fenestra, the lateral surface is dorsoventrally concave. An anteroposteriorly extending line of nutrient foramina occurs within this concavity: these begin anterodorsal to the first tooth, and at least eight are visible on the left maxilla (Fig. 2A, C). The foramina generally increase in size posteriorly, with notably large foramina above teeth 4 and 6. The number, sizes and positions of nutrient foramina vary between the right and left maxillae and are not symmetrical.

Anterior to the first tooth, the ventral edge of the maxilla arches dorsally and forms a short (but anteriorly broken) wedge-shaped anterior process, the lateral surface of which is depressed relative to the lateral surface of the main body of the maxilla (Fig. 2A, C: dia). This anterior process represents the contribution of the maxilla to an arched diastema between the maxillary and premaxillary tooth rows (the process is also visible on the anterior fragment of the right maxilla). A large foramen occurs on the lateral surface of this process on both maxillae.

Posterodorsal to the diastema is the broken base of the ascending process of the maxilla (Fig. 2A, C, H, J: asc). Above the second tooth, the base of the ascending process splits into two branches, with the lateral branch forming the damaged ventral margin of the external antorbital fenestra, and the medial branch forming the medial wall (also broken) of the antorbital fossa. Between the ventral margin of the external antorbital fenestra and the medial wall of the antorbital fossa, the antorbital fossa is deeply excavated into the body of the maxilla. This excavation, visible only in dorsal view, has a subtriangular outline (with the apex of the triangle directed medially), reaching a maximum transverse width dorsal and medial to tooth 4 (Fig. 2H, J). A large, sediment-filled, dorsomedially facing foramen opens in the posterolateral corner of the antorbital fossa (visible in dorsal and medial views), dorsal to tooth 6 (Fig. 2H: for). Anteromedial to the antorbital fossa is a medially extending maxillary shelf, the anteromedial margin of which is grooved for articulation with the opposing maxilla or another palatal bone (Fig. 2: mxsh). A palatal fragment (possibly part of the vomer) is attached by sediment to the medial surface of the maxilla (Fig. 2). The nature of the contacts with the surrounding bones (lacrimal, jugal, nasal, premaxilla) is unknown.

All of the crowns of the holotype left maxilla are damaged, with those of teeth 5 and 6 being the best preserved (Fig. 3). The mesiodistal length and labiolingual width of the erupted crowns increases to a maximum in teeth 5 and 6, with the crown of tooth 1 being considerably smaller than those positioned more posteriorly. The crown of the replacement tooth positioned medial to functional tooth 2 and visible in CT sections (see below) is approximately the same size as the more posterior ones (Fig. 3). All of the crowns are expanded at their base both mesiodistally and labiolingually: the basal expansion is similar on labial and lingual surfaces. The apex of the crown is slightly offset lingually, so the crowns are slightly asymmetrical in mesial or distal views. Coarse denticles occur along the mesial and distal edges (a denticle count is not possible due to incomplete preservation), and extend over at least 50% of the crown, rather than being limited to the apical third as in all other heterodontosaurids. The mesial- and distalmost denticles are each supported on both labial and lingual surfaces by a thickened ridge that merges with the basal expansion (‘cingulum’). Numerous subtle apicobasally extending lineations occur on the labial and lingual crown surfaces, although distinctly raised ridges are absent. Packing of the crowns is difficult to judge due to their incomplete preservation, but those of teeth 5 and 6 have a small point contact with one another (Fig. 3F). The roots of the teeth are elongate and tapering, and are inclined anterodorsally: the bases of the roots of teeth 3–5 are visible within the lateral part of the antorbital fossa. They have a subcircular cross section and are composed of a large pulp cavity surrounded by a thick layer of dentine/enamel (Fig. 3H). Towards the crown the roots become transversely compressed. Most of the area medial to the tooth row is obscured by the palatal fragment, so the presence or absence of replacement foramina cannot be assessed. However, a replacement tooth is clearly visible anteromedial to and in contact with tooth 2, although no details of its anatomy can be determined based upon external anatomy.

CT data reveal the internal anatomy of the left maxilla of the holotype in high fidelity (Figs 2, 3). The crown of the replacement tooth positioned anterodorsal to functional tooth 2 has a triangular outline in labial or lingual views, with coarse denticles visible (most clearly along the distal edge) (Fig. 3A–C, E). The root of the tooth remains broadly open at its base. The pulp cavity is proportionally larger in the erupting replacement tooth than in fully erupted teeth, with only a thin layer of dentine/enamel, which becomes even thinner towards the base of the root. Most of the root of functional tooth 2 (which is in the process of being replaced) has been resorbed. A second replacement tooth occurs medial to functional tooth 6 and is visible only in CT sections and in segmented data (Figs 2, 3). This replacement tooth is less well developed than that associated with functional tooth 2, and consists only of a crown. The root of functional tooth 6 is correspondingly less completely resorbed. This replacement tooth reveals that approximately 6 denticles occur along both mesial and distal edges of the maxillary crowns (Fig. 3D). No evidence of additional replacement teeth can be observed.

CT data also reveal the courses of the various foramina that pierce the external surface of the maxilla (Fig. 2). The foramen on the lateral surface of the anterior process opens into the posterodorsally extending dorsal alveolar neurovascular canal. Dorsal to the first tooth the canal becomes more-or-less horizontal and extends dorsal and lateral to the first four tooth roots (Fig. 2E, F, L). The canal is positioned slightly dorsal to the line of external nutrient foramina, and all of these external foramina (with the exception of the most posterior one) connect to the canal via short posterodorsally extending channels. The dorsal alveolar neurovascular canal is in close proximity to the highly porous and presumably vascular bone around the tooth roots (Fig. 3G, H). In extant crocodilians the dorsal alveolar neurovascular canal contains the nerve, arteries and veins. The larger posteriorly positioned nutrient foramen extends into an anterodorsally arching canal that is visible within the broken ventral margin of the external antorbital fenestra.

The left maxilla of LACM 128258 contains seven alveoli, with incomplete teeth in alveoli 1 and 3–6 (Fig. 7C–H). The element is badly damaged and most of the external surface of the bone above the tooth row has been lost. The available morphology is consistent with that of the holotype. The crowns of teeth 3–5 are relatively complete and have a low triangular outline: denticles are not preserved along mesial or distal edges. As in the holotype, the most mesial crown is considerably smaller than those positioned more distally. A replacement tooth is visible in CT data medial to alveolus 2 (Fig. 7C, D), but no other replacement teeth are observed.

An arched and recessed diastema between the premaxilla and maxilla occurs in other heterodontosaurids, including *Heterodontosaurus* [13], [14], “*Lanasaurus scalpridens*" [20], *Abrictosaurus* ([19]; NHMUK RU B54] and *Tianyulong* [30], although the condition for this character is uncertain in *Echinodon* and the recess is reportedly absent in *Manidens* [41]. A similar recess is absent in most other ornithischians, with the exception of some pachycephalosaurs [99], [112]. The extensive border of the external antorbital fenestra preserved in *Fruitadens* indicates the presence of a large and deeply excavated antorbital fossa, similar to that of *Heterodontosaurus* [13], [14], “*Lanasaurus*" [20], *Abrictosaurus* (NHMUK RU B54), and *Tianyulong* [30], as well as the basal ornithischian *Lesothosaurus* [33], some basal ornithopods [107], and most ornithischian outgroups. By contrast, the antorbital fossa is typically reduced in size in ceratopsians [49] and pachycephalosaurs [99], as well as most thyreophorans and derived ornithopods.

A weak buccal emargination similar to that of *Fruitadens* also occurs in *Abrictosaurus* (NHMUK RU B54), *Echinodon* [37], and *Tianyulong* [30], as well as the basal ornithischian *Lesothosaurus* and the basal thyreophoran *Scutellosaurus* [32], [33], [37], [60], [103]. By contrast, a well-developed buccal emargination occurs in many other ornithischians [113], including the heterodontosaurids “*Lanasaurus*" [20], *Manidens* [41] and *Heterodontosaurus*. The buccal emargination is particularly well developed in the latter two taxa, in which it is demarcated dorsally by a sharp ridge that defines the ventral margin of the external antorbital fenestra [13], [14], [41]. Unlike the condition in *Echinodon* [37], [39], there is no caniniform tooth in the maxilla of *Fruitadens*.

The maxillary crowns of *Fruitadens* are distinct from those of other heterodontosaurids (Fig. 8) and locally autapomorphic within the clade, and are generally reminiscent of the crowns of basal ornithischians such as *Lesothosaurus* [33] and *Scutellosaurus* [103]. They differ from the crowns of *Heterodontosaurus* (SAM-PK-K337, SAM-PK-K1332; [13], [14], [18]) in being low and triangular in labial or lingual view, being expanded mesiodistally and labiolingually above the root, in apparently having enamel evenly distributed on labial and lingual surfaces, in lacking extensive and systematically developed wear facets caused by tooth-on-tooth wear, in lacking primary ridges, in possessing denticles extending over more than 50% of the crown, and in being less tightly packed along their length (gaps remain between adjacent crowns). Similar characters separate the maxillary crowns of *Fruitadens* from those of *Abrictosaurus* [19] (NHMUK RU B54), “*Lanasaurus*" [20], NHMUK RU A100 [17], and *Lycorhinus* [21], although all of these taxa lack the sharp primary ridge seen in *Heterodontosaurus*. “*Lanasaurus*", NHMUK RU A100, and *Lycorhinus* are similar to *Fruitadens* in possessing crowns that are expanded labiolingually above the root [21]. The maxillary crowns of *Manidens* [41] have not yet been described. The maxillary crowns of *Tianyulong* [30] are similar to those of *Fruitadens* in being low and subtriangular, lacking primary ridges, lacking systematic wear facets, and not being closely packed, but differ by reportedly lacking denticles on mesial and distal crown edges [30]. The ‘cheek’ teeth (maxillary and dentary crowns) of *Fruitadens* also resemble those of *Echinodon* in that they exhibit low, triangular crowns that are expanded above the roots, lack primary ridges and systematic wear, feature symmetrically-distributed enamel, and are widely-spaced compared to the ‘cheek’ teeth of *Heterodontosaurus* [37]. However, the maxillary crowns of *Fruitadens* differ from those of *Echinodon* primarily in the possession of mesial and distal denticles that extend over more than 50% of the apicobasal height of the crown (rather than the apical third) and possessing subtle apicobasally extending lineations on labial and lingual crown surfaces [38], [39]. The possession of mesial and distal denticles extending over more than 50% of the apicobasal height of the crown appears to be autapomorphic for *Fruitadens* within Heterodontosauridae, although it may represent a retained plesiomorphy at the level of Ornithischia.

Both *Fruitadens* individuals for which skull material is preserved and has been CT-scanned show evidence of active tooth replacement; in contrast, of the five known specimens of *Heterodontosaurus*, only one (SAM-PK-K1334) shows unambiguous evidence of replacement [14].

#### Dentary

The right dentary of the holotype is the most complete, containing nine alveoli (with the anterior tip of a tenth alveolus at the posterior end) but lacking the anterior tip (including the articular surface for the predentary, assuming that this element was present as in all other ornithischians [50], [59]) and the posterior and posteroventral regions of the element (Fig. 4; supplementary video). The left dentary of the holotype is represented only by the anterior end (alveoli 1–4), although a greater proportion of the symphyseal region is preserved than in its counterpart.

The holotype right dentary possesses a curved ventral margin in lateral view that increases in dorsoventral depth posteriorly. Although the point of maximum depth is unknown due to the incompleteness of the posteroventral region, it is clear that the dorsoventral depth of the posterior dentary is substantially greater than that of the anterior dentary (this morphology is also evident in right dentary of LACM 128258). The posterior end of the right holotype dentary (as well as both LACM 128258 dentaries) is upturned, suggesting the presence of a substantial coronoid eminence. The lateral surface of the dentary is pierced by numerous nutrient foramina. The foramina are placed at irregularly spaced intervals in an anteroposteriorly extending line, ventral to the tooth row (Fig. 4A, C). The most anterior of these foramina is slightly enlarged and communicates with an anteriorly extending channel (Fig. 4A, C: adf): this foramen is probably equivalent to the ‘anterior dentary foramen’ noted by Sereno [33] in *Lesothosaurus diagnosticus*. Three additional foramina pierce the anteroventral region of the lateral surface more ventrally on the right dentary. The number, position and size of the nutrient foramina on the left and right dentaries are asymmetrical. The lateral surface of the dentary is generally convex dorsoventrally, but the tooth row is not inset at its anterior end or at the level of tooth 9. Between teeth 6 and 8 the tooth row is very slightly inset, to an even lesser extent than in the maxilla. In lateral view the tooth row is straight. Medially, the dentary is flat to gently convex dorsoventrally beneath the tooth row. Ventrally, the Meckelian groove is dorsoventrally narrow, and becomes very shallow towards its anterior termination, fading out at a point approximately level with the caniniform (Fig. 4B, D). Ventral to the Meckelian groove, beneath the gap between teeth 3 and 4, there is an elliptical foramen (with the long axis of the ellipse oriented anteroposteriorly: Fig. 4B, D, L): the Meckelian groove curves dorsally over this foramen. A similar foramen has not been described in other ornithischians, including other heterodontosaurids, and may be autapomorphic for *Fruitadens*.

The small symphyseal region is positioned anteroventrally, with its dorsal margin marked by a low curved ridge (Fig. 4B, D, L). At its anterior end (anterior to the inferred position of tooth 1) this ridge is nearly horizontal; ventral to tooth 1 the ridge curves posterodorsally, fading out ventral to the anterior part of the caniniform (tooth 2). This ridge is positioned about two thirds of the way down the bone, so that the symphysis is limited to the ventral third of the element. Ventral to the ridge, the symphyseal surface is divided into anterior and posterior concavities, which are separated by a saddle-like convexity (Fig. 4B, D, L). A small foramen occurs dorsally within the anterior concavity. Dorsal to the ridge and the symphyseal region, the medial surface of the anterior end is depressed and covered by a large oval concavity. The symphysis is not developed into a medially directed ‘spout-like’ process as occurs in most ornithischians [59]. The contact between the dentary and the predentary is not preserved in any specimen.

Parts of teeth are preserved in eight of the nine tooth positions (teeth 2–9) of the holotype right dentary, but in all cases the crowns are entirely, or almost entirely, missing. The second tooth position has an alveolus that is expanded transversely and anteroposteriorly, and the crown of the tooth (tooth 2) contained within it was presumably caniniform (see below), although only the root of this tooth is preserved (Fig. 4). The caniniform did not exceed the maximum mesiodistal and labiolingual diameters of the largest post-caniniform teeth (crowns of teeth 6–7). CT data show that the root of the caniniform was compressed transversely with an oval outline (Fig. 4M, N), and extended posteroventrally at a distinct angle to the alveolar margin, with the root tapering mesiodistally and labiolingually towards its base and reaching almost to the ventral surface of the dentary. It extends below the root of tooth 3.

Anterior to the caniniform there is a pit with an oval (right dentary: Fig. 4G, I), or circular (left dentary), outline, which (by comparison with LACM 128258; see below) probably represents an additional small alveolus (for tooth 1). CT data show no trace of a tooth in this alveolus, and that the alveolus is relatively shallow. This first alveolus is separated by a short bony margin from the anterior end of the element (as preserved) on the left side: a short diastema therefore separated the predentary from the first dentary tooth.

No diastema occurs between the caniniform and tooth 3. Tooth 3 is similar in size to the alveolus of tooth 1, but is less than 50% of the anteroposterior length of teeth 4–10. Tooth 3 has a root that extends for less than 50% of the depth of the dentary. Posterior to tooth 3, the remaining teeth increase in size to a maximum in tooth 6, and then decrease in size, with tooth 9 being similar in size to tooth 5 (based upon alveolar dimensions). Although the dentary is broken posterior to tooth 9, the cross-section of the break indicates the presence of at least one additional tooth, meaning that the dentary tooth count in the holotype was at least 10.

All of the erupted crowns are badly damaged (Fig. 4). The crowns are expanded mesiodistally and labiolingually above the root. This expansion is similar on labial and lingual sides. On the lingual side of the crown of tooth 6, basal ridges that connect to the mesial and distal most denticles are evident. A similar, weakly developed ridge is visible on the distal margin of the labial surface of this crown, but it is unclear if a mesial ridge occurs. Several subtle apicobasally extending ridges are visible on the lingual surface of the base of the crown of tooth 7. The crowns are moderately closely packed (adjacent alveoli are continuous with one another), and would likely have slightly overlapped one another or at least contacted one another. The roots of the crowns are transversely compressed with oval cross-sections and are vertically oriented (Fig. 4H). Each tapers towards its base and has a broad pulp cavity that is surrounded by a thick layer of dentine/enamel (Fig. 4M), and each root generally extends for about two-thirds of the dorsoventral height of the dentary (Fig. 4E, F).

Clear evidence of tooth replacement is visible in the right dentary. Medial to the tooth row, a thin strip of bone separates the alveoli from the medial surface of the dentary. Slit-like replacement foramina are visible within this bony strip ventromedial to the crowns of teeth 5 and 8 (Fig. 4B, D). These teeth appear to be among the most heavily erupted (i.e. a large section of the root is visible beneath the crowns). This bony strip is absent beneath the crown of tooth 6, which is the most recently erupted (none of its root is visible). The crown of tooth 9 is in the process of replacement: only a very small fragment of the erupted crown is preserved, and a replacement crown is partially visible in medial view. This replacement crown has coarse denticles along mesial and distal surfaces, and the mesial and distal most denticles are particularly pronounced and supported by ridges (Fig. 4H, K). CT data provide additional information on tooth replacement, with the three replacement teeth clearly visible (Fig. 4H). The replacement teeth are positioned medial and slightly posterior to the functional tooth they are replacing. The replacement teeth for functional teeth 5 and 8 consist only of partial crowns, with coarse denticles along mesial and distal surfaces, and the roots of functional teeth 5 and 8 are partially resorbed. Replacement teeth are not observed elsewhere in the dentary.

As in the maxilla, CT sections clarify the courses of the foramina that pierce the external surface of the dentary (Fig. 4E, F, J). The ‘anterior dentary foramen’ opens into the mandibular canal that runs within the dentary, ventral and lateral to the tooth roots. All other foramina positioned on the lateral, ventrolateral and medial surfaces of the dentary connect to the mandibular canal via smaller channels. The mandibular canal extends posteriorly lateral to the root of the caniniform. Ventral to tooth 4 it expands in size and becomes more centrally positioned, close to the ventral margin of the dentary. The canal continues at least as far as tooth 7; beyond this point the posteroventral margin of the dentary is broken away. The canal very closely approaches the bases of the tooth roots, and in some cases communicates with the space surrounding the roots. The canal contained the inferior alveolar nerve as well as vasculature.

As mentioned above, the dentaries of LACM 128258 (Figs. 5, 6, supplementary video) are damaged, and it is not possible to readily determine many anatomical details but their morphology is generally consistent with that of the holotype. Originally a caniniform crown was preserved in the right dentary and it is shown in photographs and drawings made in the 1980s (Fig. 5L). Unfortunately this crown has since been lost; however, a cast of the jaws of LACM 128258 as originally recovered contains the caniniform (Fig. 5N). The cast shows that the caniniform was recurved, and the erupted height of the crown was subequal to the maximum height of the largest post-caniniform crown (that of tooth 7). The labial surface of the caniniform was strongly convex mesiodistally, whereas the lingual surface was more weakly convex. It is not possible to determine the presence or absence of serrations on the mesial and distal edges of the caniniform.

The right dentary of LACM 128258 contains 10 teeth (teeth 2–4 represented by the roots only) and there is a possible alveolus of an eleventh tooth visible in CT scans. The root of the caniniform (tooth 2) is present but the tooth is broken at the level of the oral margin (Fig. 5). As in the holotype, the root is transversely compressed with an oval outline: the labiolingual diameter is approximately two-thirds of the mesiodistal length. The large pulp cavity is surrounded by a thin layer of dentine/enamel. The root of the caniniform tapers posteroventrally, although at a shallower angle to the vertical than in the holotype, and approaches the ventral margin of the dentary (Fig. 5E, F). The mesiodistal length of the root of the caniniform is less than 80% of the length of the largest root (that of tooth 7).

Anterior to the caniniform, and separated from it by a short gap, there is an additional tooth (tooth 1), with the root and most of the crown (with the exception of the tip) preserved (Fig. 5K, M). Tooth 1 is small with a mesiodistal length of the crown of less than 1 mm, approximately 35% of the largest crown (tooth 7) in the dentary and just over 50% of the length of the caniniform. The crown of tooth 1 is slightly labiolingually compressed, with the labiolingual diameter approximately 75% of the mesiodistal length. The crown is slightly procumbent, with convex labial and lingual surfaces, and has a root that extends posteroventrally for less than half of the depth of the dentary. The crown is not expanded either mesiodistally or labiolingually above the root. In labial or lingual view the distal margin of the crown is convex and lacks serrations or denticles; the mesial surface is not exposed. The pulp cavity is large with a thin external layer of dentine/enamel.

Tooth 3 (missing, root visible in CT scans: Fig. 5K, M) is positioned immediately adjacent to the caniniform. The alveoli of teeth 2 and 3 are confluent and there is no post-caniniform diastema. Tooth 3 is very short in mesiodistal length, being less than a third of the length of the caniniform. Its vertically oriented root extends for about a quarter of the depth of the dentary. Tooth 4 is also broken and represented by the root only; the root is relatively small, with a mesiodistal length similar to that of tooth 1 and substantially shorter than the caniniform or more posteriorly positioned teeth (Fig. 5K, M). As in the more posterior crowns, the root of tooth 4 is compressed labiolingually, with the labiolingual diameter approximately 70% of the mesiodistal length. The pulp cavity is small, with its diameter being similar to the thickness of the external dentine/enamel layer. The root extends anteroventrally for about half of the depth of the dentary.

The remaining teeth have at least partially preserved crowns, with the crowns of teeth 6–10 being relatively complete. They are all substantially larger than tooth 4. The teeth increase in size to a maximum in tooth 7, then decrease in size, with the last tooth being similar in size to tooth 5. Their roots are transversely compressed, with labiolingual widths that are approximately two-thirds of the mesiodistal lengths, and possess large pulp cavities. The roots, which taper towards their bases, extend nearly to the ventral margin of the dentary and show a variety of orientations: the roots of teeth 5–6 and 9–10 are directed anteroventrally, those of teeth 7–8 are nearly vertical.

The crowns of teeth 7 and 8 contact one another at their broadest part but they do not overlap, and no imbrication occurs elsewhere, with adjacent crowns being separated from one another by small gaps. The crowns have a low triangular shape in labial or lingual views, and are expanded labiolingually and mesiodistally above the root. The apex of the crown is offset slightly lingually. Well-developed ridges connect the mesial and distal most denticles to the basal expansion, and coarse denticles occur along mesial and distal edges. These denticles are unfortunately damaged and an accurate count is not possible. Some subtle apicobasally extending ridges extend along the lingual crown surfaces. Replacement teeth are visible medial and posterior to teeth 1, 6 and 10 in CT data (Fig. 5M).

The left dentary of LACM 128258 (Fig. 6) preserves the posterior half of the root of the caniniform (tooth 2). The root of a very small tooth (tooth 3) is preserved immediately adjacent to the caniniform, and is then followed by a gap before the second preserved postcaniniform (tooth 5): the position of this gap corresponds to the fourth tooth of the right dentary. Six large teeth occur posterior to this gap so that the total preserved tooth count is ten (teeth 2, 3 and 5–10 are present; teeth 1 and 4 are missing. Two replacement teeth are visible medial to teeth 6 and 10 in CT data (Fig. 6K). A small fragment of bone attached to the medial surface of the dentary at its posterodorsal tip may represent part of the coronoid.

LACM 128303 represents the anterior end of the left dentary of an individual about 60% of the size of the holotype (Fig. 7I, J). The anterior four tooth positions are empty, whereas crowns are preserved in tooth positions 5–9. The dentary is broken anteriorly at the level of the first alveolus, ventrally and posteriorly. Part of the dentary symphysis is preserved. The relative sizes of the empty alveoli 1–4 suggest a similar pattern of dentition to other specimens, with a small tooth 1, an enlarged tooth 2 (the caniniform), a small tooth 3, and then a larger tooth 4. The crowns of teeth 5–9 are poorly preserved, but generally similar to those of other *Fruitadens* dentaries: they are low, subtriangular, expanded mesiodistally and labiolingually above the root, with coarse denticles, and apicobasally extending ridges occur on the lingual crown surface.

The dentary of *Fruitadens* strongly resembles that of *Echinodon* (NHMUK OR 48213, 48215a, 48215b), which also tapers anteriorly, possesses a strongly upturned posterior end, lacks a well-developed buccal emargination, and possesses an anterior dentary foramen associated with an anteriorly extending groove [37]. Moreover, although the dentaries of *Echinodon* are transversely crushed, the form of the symphyseal area (NHMUK OR 48215b) is essentially identical to that of *Fruitadens*. By contrast, the dentary of *Heterodontosaurus* is slightly dorsoventrally expanded at its anterior end, has a nearly straight ventral margin, a deep buccal emargination, the anterior dentary foramen is not associated with a laterally positioned groove, and the symphyseal surface is not restricted to the ventral third of the element [14]. The dentaries of *Tianyulong* and *Abrictosaurus* are also slightly expanded at their anterior ends and do not taper anteriorly [19], [30]. Both have weak buccal emarginations similar to that of *Fruitadens*. The dentary of *Manidens* appears to be much more robust than that of *Fruitadens* [41].

The presence of a dentary caniniform is a common heterodontosaurid character that is absent in almost all other ornithischians, with the exception of some, but not all, pachycephalosaurs [112] and which is also absent in *Echinodon* [37] and *Abrictosaurus* [19]. An enlarged caniniform tooth positioned anteriorly within the dentary and a ‘subnarial gap’ occurs in basal theropods [61], [114], and the possible homology of this character with the dentary caniniform of heterodontosaurids is worthy of future examination. The dentary caniniform of *Fruitadens* differs from those of *Heterodontosaurus* [14], *Lycorhinus* [21], NHMUK RU A100 [17], and *Tianyulong* [30] in being relatively short apicobasally, and not exceeding in size the largest postcaniniform teeth. The presence of an apicobasally short caniniform appears therefore to be autapomorphic for *Fruitadens* within Heterodontosauridae, although the caniniform of *Manidens* may also be relatively short [41]. Moreover, the dentary caniniform of *Heterodontosaurus* further differs from those of *Fruitadens* and *Tianyulong* [30] in being less strongly recurved [14].

The presence of an additional tooth anterior to the dentary caniniform is a unique character of *Fruitadens*, being absent in all other heterodontosaurids [13], [14], [21], [30] and other ornithischians that possess a dentary caniniform [112], although two additional teeth occur anterior to the similar enlarged anterior caniniform of basal theropods [114]. Interestingly, this additional tooth in *Fruitadens* is only preserved in the smaller dentary (LACM 128258) and the first alveolus is empty on both sides in the larger holotype (LACM 115747) despite the fact that teeth (or at least tooth roots) are preserved in all other tooth positions. Further specimens are required to determine the significance of this observation with regard to sexual dimorphism, individual variation and ontogeny: e.g. is it possible that the ‘pre-caniniform’ occurs only in early ontogenetic stages of *Fruitadens* and is later lost?

The morphology of the postcaniniform dentary teeth (i.e. the ‘cheek’ teeth) is highly similar to that of the maxillary teeth, and the same characters that distinguish the maxillary teeth of *Fruitadens* from other heterodontosaurids also distinguish the dentary teeth (Fig. 8). The dentary teeth of *Manidens* are highly distinct from those of *Fruitadens* in being apicobasally tall and (in more distal crowns) highly asymmetrical in labial view, with subdivided denticles [41].

#### Vertebral column: general comments

The vertebrae of the holotype are disarticulated and there is no information concerning their preserved positions. Callison (in [115]: 13) incorrectly suggested that the neural arches were separated from the centra prior to burial; parts of the neural arch are in fact preserved in all elements. In every vertebra (with the exception of the caudal vertebrae, in which the neural arch and centrum are indistinguishably fused) the neurocentral sutural line is visible as a thin line of matrix, so these elements are not fused together.

#### Cervical vertebrae

The most complete cervical in the holotype is probably from the anterior end of the postaxial column (Fig. 9A–D), based upon the wedge-shaped centrum, with the two articular ends converging ventrally in lateral view, and the posterior one offset ventrally. As preserved, this vertebra lacks the zygapophyses, neural spine and the articular ends of the parapophyses and diapophyses. The roof of the neural arch is almost flat, and the centrum is pinched in transversely with a ventral median ridge that is not clearly delimited from the rest of the centrum. The flat articular ends of the centrum are subtriangular in outline. The neural canal is proportionately large, being as broad as the centrum. The parapophysis is positioned anterodorsally upon the lateral surface of the centrum, and the diapophysis is positioned at the midpoint of the neural arch. Although the postzygapophyses are broken at their bases, a faint ridge on the base of the dorsal surface of the right postzygapophysis may represent an epipophysis.

One additional cervical vertebra occurs in the holotype (probably from the posterior end of the cervical column, possibly cervical 7 or 8), but the majority of the neural arch has broken away above its base and only the centrum is well preserved (Fig. 9E, F). The centrum has a prominent parapophysis on its anterodorsal margin, positioned mostly on the base of the neural arch but also extending onto the neurocentral suture. The centrum has articular ends that converge dorsally in lateral view. The anterior face of the centrum is diamond shaped and flat to slightly convex; the posterior face is triangular in outline and flat to slightly concave. The lateral surfaces of the centrum are strongly pinched inwards with one large nutrient foramen (as well as multiple smaller foramina) on each surface. A well-developed, keel-like, ventral ridge is transversely narrow and sharp edged posteriorly, and becomes progressively wider and more rounded towards its anterior end (Fig. 9F). The ventral margin of the centrum in lateral view is strongly convex (Fig. 9E).

The cervical vertebrae are generally similar to those of small-bodied ornithischians, including *Heterodontosaurus* (SAM-PK-K1332; [11]) and *Hypsilophodon* [106]. The anterior cervical vertebra of *Fruitadens* compares well with cervical 3 of these taxa, sharing with them the transversely pinched centrum and the ventrally convergent and offset articular faces. The posterior cervical centrum of *Fruitadens* is similar in its proportions to the posterior cervical centra of *Heterodontosaurus* and *Hypsilophodon*; however, the posterior cervicals of these taxa differ in possessing concave-to-flat ventral margins in lateral view. Convex ventral margins of cervical centra are seen in several other ornithischian taxa, including the small ornithopods *Changchunsaurus*, *Jeholosaurus*, and *Othnielosaurus*, and the basal ceratopsian *Chaoyangsaurus* [116].

#### Dorsal vertebrae

Two isolated centra in the holotype are reminiscent of cervical vertebrae in possessing moderately pinched lateral surfaces and a ventral keel. No trace of a parapophysis on occurs on the anterodorsal part of either centrum, and it is therefore likely that these vertebrae represent anterior dorsals. The anterior articular face in both is flat to slightly convex, whereas the posterior surface is flat to slightly concave. One of these centra possesses a laterally compressed ventral projection at its anterior end that extends 2 mm ventral to the anterior articular surface (Fig. 9G, H). In the second centrum the ventral keel is broken. Tiny nutrient foramina occur on the lateral surfaces of both centra.

A third anterior dorsal vertebra in the holotype lacks the diapophyses, most of the neural spine and zygopophyses, and the anteroventral corner of the centrum (Fig. 9I–K). The posterior articular face is wider than deep and gently concave. The lateral surfaces of the centrum are gently concave anteroposteriorly (not strongly pinched), and ventrally there is no midline keel. Nutrient foramina occur on the lateral surfaces of the centrum. The parapophysis is mainly positioned on the anteroventral part of the neural arch, but also extends slightly onto the anterodorsal corner of the centrum. The neural canal is broader than deep, with an oval outline (Fig. 9I). The base of the neural spine is elongate, extending for the full length of the neural arch. The left postzygapophysis faces lateroventrally at approximately 45° to the horizontal. Another incomplete dorsal vertebra in the holotype has a centrum with a very similar morphology, but there is no trace of the parapophysis on the preserved part of the neural arch.

There are two additional isolated centra in the holotype, both with a very small part of the neural arch attached (Fig. 9L, M). These centra are the same light grey to slightly brownish color as the anterior section of the sacrum, so they were probably preserved adjacent to it, and may represent the last two dorsal vertebrae. The centra are spool-shaped, lacking ventral keels, with lateral surfaces that are gently concave anteroposteriorly. The articular ends are broader than deep, and are flat to slightly concave.

A single poorly preserved dorsal vertebra occurs in LACM 128258. It has a spool-shaped centrum, with no trace of a ventral keel, and gently concave articular faces that are broader than deep. The neural canal is large, being as deep and nearly as broad as the centrum. The transverse processes, prezygapophyses and the neural spine are all damaged and incomplete. The base of the neural spine is elongate, extending for the full length of the neural arch. The elongate postzygapophyses extend beyond the posterior margin of the centrum and have articular faces that face ventrolaterally at around 10° to the horizontal.

The fragmentary nature of the dorsal column of *Fruitadens*, and the extremely limited data available on the dorsal column of other heterodontosaurids (even in *Heterodontosaurus*, in which the complete dorsal column is not well exposed [11]), prevents detailed comparisons, although the dorsal vertebrae appear to be generally similar to those of other small-bodied ornithischians. One unusual feature is the ventral projection on the anterior end of the centrum of one of the anterior dorsals. A similar feature occurs in the anterior four dorsal vertebrae of the basal ornithopods *Changchunsaurus* and *Jeholosaurus* [116], but appears to be absent elsewhere within Ornithischia (including *Hypsilophodon*), although the condition for this character is unknown for most other heterodontosaurids. The ventral surfaces of the centra are generally not exposed in the dorsal vertebrae of *Heterodontosaurus* described by Santa Luca [11], with the exception of the first dorsal which appears to lack a ventral projection.

#### Sacral vertebrae

The sacrum of the holotype is preserved in three parts (Fig. 10). The first is an isolated centrum (with a very small attached part of the neural arch), similar in morphology to the posterior dorsals discussed above (i.e. spool-shaped with gently excavated lateral surfaces) (Fig. 10A–D). Two features suggest that this centrum formed part of the sacrum. First, the posterior part of the centrum is deeply excavated by the neural canal (Fig. 10D), in a manner similar to that seen in the other sacrals (see below), and this excavation becomes much shallower anteriorly (Fig. 10C). Second, the posterior articular face of the centrum has a radiating pattern of ridges and grooves, similar to that seen in the other sacrals (Fig. 10D).

The second part of the sacrum consists of centra 2–4 (Fig. 10E–G), with only fragments of the neural arches preserved. The anterior face of sacral 2 has a U-shaped outline, with the dorsal surface deeply excavated by the neural canal (Fig. 10F). The articular face has a series of ridges and grooves, similar to those of sacral 1. The centrum of sacral 2 is expanded transversely at its posterior end to help support the massive sacral rib of sacral 3 (Fig. 10G). The sinuous suture between sacral centra 2 and 3 (and the similar suture between sacral centra 3 and 4) is visible, so the elements are not indistinguishably fused. A fragment of the left postzygapophysis is preserved in articulation with (but not fused to) the prezygapophysis of sacral 3, but the rest of the neural arch of sacral 2 is missing. Sacral 3 has a centrum with a flattened ventral margin and a subtle midline ridge, and bears a massive, posterolaterally directed sacral rib on each lateral surface (Fig. 10E–G). These ribs attach to almost the entire lateral surface of the centrum as well as to the lateral surface of the neural arch. The neural arch is poorly preserved: only the left prezygapophysis is visible. The centrum of sacral 4 has a sinuous suture with sacral 3, a flattened ventral margin (with a very weak midline groove), and a deeply excavated neural canal. The posterior articular surface has a U-shaped outline, and is covered by a series of ridges and grooves. The neural arch of this vertebra is almost entirely missing and the morphology of the sacral rib is unknown.

The third part of the sacrum consists of two articulated vertebrae (Fig. 10H–J). The first of these sacrals (sacral 5) has a centrum with a U-shaped anterior articular face, a deeply excavated neural canal, and a slightly flattened ventral surface. The anterior articular face has a pattern of ridges and grooves that appears to match that of the posterior surface of sacral 4, suggesting that the two were originally in articulation. A large laterally directed sacral rib is preserved on the left side, borne on the anterodorsal corner of the centrum, and partially supported by the preceding sacral. This sacral rib does not appear tobe present on the right side: instead, a massive sacral rib contacts the posterodorsal end of the centrum (Fig. 10J), as well as most of the lateral surface of the neural arch of the succeeding centrum (sacral 6). The morphology of the sacrum is therefore asymmetrical in this region, although it is possible that the right side is damaged, with the sacral rib being posteriorly displaced. Sacral 6 has a smooth and gently concave posterior articular face (Fig. 10H) with an oval outline (being wider transversely than dorsoventrally deep), and the neural canal is small and does not extensively excavate the centrum. The neural arch is incomplete but carries a large sacral rib on each lateral surface.

Information on the sacra of other heterodontosaurids is limited to a single specimen of *Heterodontosaurus* in which the sacrum is largely hidden by the articulated ilia [11], the sacrum of *Manidens*, which is only briefly described [41], and the incompletely prepared postcranium of the holotype of *Abrictosaurus* (NHMUK RU B54). *Fruitadens* is similar to *Heterodontosaurus* [11], *Manidens* [41], most basal ornithopods [106], and marginocephalians [99], [101] in possessing six sacral vertebrae. By contrast, basal ornithischians such as *Lesothosaurus* [33] and *Agilisaurus* [105] possess five sacral vertebrae, and five sacrals may also occur in *Abrictosaurus* (NHMUK RU B54). Although *Heterodontosaurus* and *Fruitadens* possess a sacral count of six, there are differences between the sacra of these taxa and those of most basal ornithopods, ceratopsians and pachycephalosaurs. In most representatives of the latter clades, sacrals 2–6 possess large sacral ribs on the lateral surfaces of their centra that contact the main body of the ilium [99], [106] (*Archaeoceratops*, IVPP V11114). By contrast, in *Heterodontosaurus* [11] and *Fruitadens*, only four large sacral ribs attach to the lateral surfaces of the centra of sacrals 3–6. In *Heterodontosaurus*, the ribs of sacrals 1–2 are both slender and attach to the preacetabular process of the ilium [11], whereas there is only a single slender rib in basal ornithopods and this rib is attached to the preacetabular process in ceratopsians and pachycephalosaurids. These differences in morphology suggest that the evolution of six sacral vertebrae in heterodontosaurids may have occurred independently to that in ornithopods, ceratopsians and pachycephalosaurs, via the incorporation of an additional dorsal vertebra into the sacrum.

#### Caudal vertebrae

The holotype contains six incompletely preserved proximal caudal vertebrae (Fig. 11), as well as three complete or mostly complete distal caudals. Numerous additional vertebral fragments probably belong to the caudal series. Neurocentral sutures appear to be indistinguishably fused in all caudal vertebrae. A caudal vertebra with a complete and highly elongate neural spine may represent the first caudal (Fig. 11A–C), or one of the first few caudals, based on comparisons to *Heterodontosaurus* in which the neural spine of the first caudal is elongate and slender [11]. This vertebra has a centrum that is longer than deep, with gently excavated lateral surfaces. Its articular faces are broader than high, with the anterior face being gently concave to flat and the posterior face being concave. The posterior articular face is offset ventrally relative to the anterior face in lateral view. The transverse processes are broken but their bases are positioned at the approximate level of the neurocentral suture and the processes would have projected laterally. The postzygapophyses are positioned relatively high on the neural arch, are widely divergent, and have large articular surfaces that face ventrolaterally at around 25° to the horizontal. The neural spine is transversely compressed, anteroposteriorly narrow, and positioned posteriorly on the neural arch.

Other proximal caudals (Fig. 11D–G) are generally similar in morphology to the first, although their articular faces tend to be deeper and more shield-like. All of the other proximal caudals are missing their neural spines and prezygapophyses, and most lack the postzygapophyses. One proximal caudal possesses an elongate, laterally projecting transverse process that exceeds the centrum in length (Fig. 11D, E). Well-developed chevron facets do not appear to be present.

Two of the three distal caudals are very poorly preserved (Fig. 11I); the other is well preserved (Fig. 11H) and has a long and low centrum (more than 2.5 times as long as high), with articular faces that have an approximately hexagonal outline. The anterior articular face is flattened; the posterior face is concave. The ventral surface of the centrum is flat to slightly concave transversely, and is separated by an anteroposteriorly extending ridge from the lateral faces of the centrum. The lateral faces are gently concave anteroposteriorly, and are subdivided by an anteroposteriorly extending ridge positioned at midheight. Dorsal and ventral to this ridge the lateral surface is concave dorsoventrally. The anterior and posterior ends of the centrum are not beveled for articulation with chevrons. No traces of transverse processes occur and a neural spine is absent (although a very subtle and broad midline ridge extends for the entire length of the neural arch). The pre- and postzygapophyses extend only a very short distance anterior and posterior to the faces of the centrum, and have articular faces set at about 20–30° to the horizontal. A similar distal caudal is preserved in LACM 120602.

Comparisons are limited due to the incomplete preservation of the caudal vertebrae; however, all preserved caudals are closely comparable to those of *Heterodontosaurus tucki* [11] as well as other small-bodied ornithischians [106].

#### Humerus

The shaft of the almost complete left humerus of LACM 120478 (Fig. 12) has been repaired in several places; as a result the original orientation of the proximal and distal ends relative to one another is unknown. The proximal and distal ends of the element are expanded transversely, and coarse endochondral bone trabeculae are visible at both ends, as in the femur and other limb bones of this specimen. The articular head is subspherical and positioned laterally, and is set off from the prominent medial tuberosity by a groove and a constriction in proximal view (Fig. 12E). The deltopectoral crest is unfortunately missing in the specimen as currently preserved; however, earlier photographs of the specimen (Fig. 12G, H) show that the apex of the crest was positioned at about 30% of length, and was directed anteriorly and slightly laterally. The anterior edge of the crest was gently concave proximodistally in lateral view. Transversely, the anterior surface of the proximal third of the element is concave whereas the posterior surface is convex; the shaft beyond this is subcircular in cross section for most of its length. At the distal end, near the condyles, the shaft becomes expanded transversely and thus has a subrectangular cross-section. The long-axis of the medial ulnar condyle is oriented anteroposteriorly and the long-axis of the radial condyle is oriented anterolaterally-to-posteromedially (Fig. 12F). Anteriorly there is a shallow depression above the condyles on the distal end. There are no well-developed ent- or ectepicondyles on the medial and lateral surfaces of the distal end; however, there are shallow depressions on the articular surfaces of the radial and ulnar condyles (Fig. 12F).

The humerus shares with that of *Heterodontosaurus* the presence of a constriction in proximal view between the head and the medial tubercle [11]. This constriction seems to be absent in other ornithischians, and may therefore represent a heterodontosaurid synapomorphy [61]. However, the deltopectoral crest of *Fruitadens* is proportionally smaller than the well-developed crest of *Heterodontosaurus*, and the distal humerus lacks the well-developed ectepicondyle that occurs in *Heterodontosaurus* [11]. With the exception of the proximal constriction, there are no characters that clearly distinguish the humerus of *Fruitadens* from that of most other small-bodied ornithischians (e.g. *Hypsilophodon* [106]).

#### Femur

The holotype includes the proximal end of the right femur, broken distal to the base of the anterior trochanter (Fig. 13A–E); LACM 115727 contains the proximal end and most of the shaft of the right femur (Fig. 13F–K) and the proximal end (missing the head) of the left femur; and LACM 120478 includes the distal 75% (including the distal end) of the left femur (Fig. 13L–R). The proximal femora of LACM 115747 and LACM 115727 are similar in size (LACM 115747 being a slightly larger individual), while LACM 120478 is considerably smaller in size. The distal end of LACM 120478 shows longitudinally oriented coarse endochondral bone trabeculae consistent with a juvenile that is undergoing extensive longitudinal growth at the time of death [61: ESM].

The shaft is arched anteriorly along its length in lateral view (LACM 120478; Fig. 13M). The shaft ventral to the position of the fourth trochanter curves laterally, so that in anterior and posterior views the lateral surface is gently concave and the medial surface gently convex. Immediately proximal to the fourth trochanter the shaft of LACM 120478 is broken, but appears to be beginning to curve medially. In LACM 115727 the femur is arched strongly laterally along its length in anterior and posterior views, but the shaft has been broken and repaired in several positions and may not reflect exactly the original morphology. As a result, the complete femur of *Fruitadens* probably had a sinusoidal outline in anterior and posterior views.

The head is offset strongly perpendicular to the shaft in anterior and posterior views (Fig. 13A, C, F, H). In proximal view (Fig. 13E, J) the proximal end is narrowest at its medial margin (the femoral head), and expands gradually anteroposteriorly towards its lateral margin (the broad greater trochanter). In proximal view (Fig. 13E, J), the anterior surface of the proximal end is gently concave, the posterior surface is flat to gently concave, the medial surface (the head) is strongly convex, and the lateral surface (the greater trochanter) is gently concave. The transverse groove (the *fossa trochanteris* of Langer [117]) seen in basal dinosaurs is absent from the proximal surface, and there is no constriction separating the head from the proximal trochanters (in anterior and posterior views the proximal end is very gently concave transversely, but a distinct constriction or depression is absent). The proximal articular surface is strongly convex anteroposteriorly. The head possesses a weak posterior projection at its posteromedial corner in the referred specimen LACM 115727 (Fig. 13J), a feature that does not occur in the holotype (Fig. 13E). The greater trochanter is wider anteroposteriorly than the head, and it is fused with the anterior trochanter in LACM 115727 and LACM 115747 (Fig. 13B, G). The greater trochanter is thickened into a prominent ridge along its posterior edge (Fig. 13C, H: tri); this ridge separates the posterior and lateral surfaces of the proximal end. In both right and left femora of LACM 115727 there is a low knob-like projection with a roughened surface texture on the posterior margin of this ridge (Fig. 13H :pr); this region of the femur is damaged in the holotype. The lateral surface of the greater trochanter is covered (anterior to the prominent ridge described above) by a depression (Fig. 13B, G: ldp), and is concave anteroposteriorly. The anterior trochanter is narrower anteroposteriorly than the greater trochanter, is set anterolateral to the greater trochanter, and has an oval cross-section, with the long axis of the oval directed anteroposteriorly. Although fused to the greater trochanter, it can clearly be distinguished from it by a break-in-slope in lateral view, and a trace of a notch separating the trochanters is visible in proximal and anteromedial views (Fig. 13D: nt).

The fourth trochanter is not preserved in LACM 115747 (the bone surface in this area has flaked away) and only the base of the trochanter is currently preserved in LACM 120478, although older photographs and drawings of this specimen demonstrate that a rod-like fourth trochanter originally was present (Fig. 13R). The shaft of the femur immediately proximal to the fourth trochanter has a sub-circular cross-section, with a hollow shaft (Fig. 13P). The base of the fourth trochanter begins on the posteromedial edge of the shaft; the distal end of the base is positioned close to the posterolateral edge of the shaft. The base is transversely compressed, narrow, and proximodistally short. A depression occurs adjacent to the proximomedial edge of the base of the trochanter, and a longer groove-like depression adjacent to the lateral edge of the base of the trochanter. Distal to the fourth tranchanter, a sharp ridge extends along the posterolateral surface of the shaft, and connects to the lateral margin of the lateral condyle.

Distally the femur is expanded transversely and posteriorly to form the condyles. In distal view (Fig. 13Q), there is a prominent U-shaped intercondylar groove on the posterior surface, separating the medial and lateral condyles. Both medial and lateral condyles have a strongly convex outline. A transverse groove separates the lateral condyle from the prominent fibular condyle in distal and lateral views (Fig. 13M, Q: gr). The anterior and lateral surfaces of the distal end meet at an oblique angle: the anterior surface is flat to very gently concave transversely but a well-developed anterior intercondylar groove does not occur. In anterior and posterior views the fibular and lateral condyles extend very slightly further distally than does the medial condyle.

The femur closely resembles that of *Heterodontosaurus* ([11]; SAM-PK-K1332]). In both taxa there is no constriction separating the head from the greater and anterior trochanters, the greater and anterior trochanters are fused to one another, and there is a rod-like (parallel-sided) and pendant fourth trochanter. The absence of a constriction separating the head from the greater and anterior trochanters is a plesiomorphy shared with basal ornithischians such as *Lesothosaurus* [33], as well as ornithischian outgroups; more derived ornithischians typically have a well-developed constriction [106]. The fusion of the greater and anterior trochanters differs from the condition in most other ornithischians, including the heterodontosaurid *Abrictosaurus* in which the trochanters are separated by a deep cleft (NHMUK RU B54). Finally, the rod-like fourth trochanter is absent in other ornithischians [33], [106], and may represent a heterodontosaurid synapomorphy.

#### Tibia

LACM 120478 contains a complete left tibia in articulation with the left fibula, astragalus and calcaneum (Figs. 14A–F, 15A–D). The proximal and distal ends of both tibiae (as well as unidentifiable portions of the shafts of long-bones which may represent parts of the tibiae) occur in the holotype (Fig. 14G, H, N, O; separate from the astragalocalcaneum that is not preserved), although the proximal ends are damaged, while the proximal and distal ends of the left tibia occur in LACM 115727 (Figs. 14I–M, 15E–H) in articulation with the astragalocalcaneum. The bone surface of the proximal and distal ends of the tibia of LACM 120478 consists of coarse endochondral bone trabeculae similar to that of the femur, consistent with the inferred immature status of this individual. Like the femur, cross-sections show that the tibia was extensively hollowed.

The complete tibia is slender with the proximal end expanded strongly anteroposteriorly and weakly transversely, the distal end expanded weakly both transversely and anteroposteriorly, and the bone is therefore twisted through about 70° along its length. The proximal end is well preserved in LACM 120478 and the proximal surface is obliquely inclined, with the medial edge positioned more proximally than the lateral edge.

The cnemial crest has a transverse diameter that is greater than the transverse diameter of the inner condyle (Fig. 14E). The cnemial crest projects anterolaterally (but not dorsally). In proximal view, the medial surface of the proximal end is convex (Fig. 14E). On the lateral surface, the insisura tibialis, a proximodistally extending sulcus, separates the cnemial crest from the fibular condyle. The fibular condyle projects posterolaterally, and possesses a small accessory condyle on its anterolateral surface. The fibular and inner condyles are separated from one another by a deep ‘V’-shaped notch. A sharp ridge (fibula crest) extends distally from the accessory condyle and articulates with the fibula. Although incompletely exposed, this ridge appears to extend along the lateral surface for almost the entire length of the tibia: it is visible on the laterodistal part of the shaft in LACM 115727, and there is a narrow concave depression medial to it for the fibular shaft.

In the holotype the distal ends of the tibiae are preserved without the astragalocalcaneum (Fig. 14N, O). The distal end generally has a sub-rectangular outline, but at its medial margin it is drawn out into a well-developed, anteromedially directed and transversely compressed sheet (Fig. 14N, O: amsh; Fig. 15). This sheet articulated with the medial surface of the large ascending process of the astragalus. In lateral view the distal edge of the anteromedial sheet is convex, where it articulated with the proximal surface of the astragalus. Lateral to the “anteromedial sheet", the main body of the distal tibia is broader transversely than anteroposteriorly (Fig. 14N). Its posterior surface is flat to slightly concave, and its anterior surface is convex in distal view, with a low proximodistally extending ridge separating transversely concave facets for the ascending process of the astragalus (medially) and the calcaneum and fibula (laterally). A small articular surface occurs for the transversely expanded distal fibula (LACM 115727). A low ridge on the posterior surface of the distal end separates the anteromedial flange from the main body of the tibia and it can be traced up the posterior surface of the shaft to connect to the inner condyle (LACM 120478). A second ridge separates the posterior and lateral surfaces of the distal tibia. The lateral surface of the distal tibia is strongly depressed and scarred adjacent to the calcaneum. In distal view, the distal surface of the tibia is concave for articulation with the astragalocalcaneum.

As in the basal ornithischians *Eocursor* [9], *Lesothosaurus* [118], *Scutellosaurus* (MNA Pl.175), and *Hexinlusaurus* [119], proximally the inner condyle is elongate, extending posteriorly far beyond the fibula condyle, although the inner condyle is proportionally narrower in *Fruitadens* than in these taxa. By contrast, the inner condyle is relatively shorter in basal ornithopods [106] and basal ceratopsians [120]. The distal end of the tibia of *Fruitadens* and *Heterodontosaurus* is not so strongly expanded transversely as in most other ornithischians: the maximum distal width of the tibia is 15% of the length of the tibia in *Heterodontosaurus* [11] and 12% in *Fruitadens*, whereas it is 19% in *Eocursor* [7], 21% in *Hexinlusaurus* [119], 22% in *Hypsilophodon* and *Othnielosaurus* [106], [121] and 31% in *Psittacosaurus sibiricus* [120], although it is possible that this character is in part size-related. A particularly unusual character of the distal tibia of *Fruitadens* is the presence of an anteromedially extending, transversely compressed flange that articulated with the medial surface of the ascending process of the astragalus. Such a flange is absent in other ornithischians, in which the articular surface for the astragalus faces almost entirely anteriorly ([9], [106], [119]; *Scutellosaurus*, MNA P1.175). Moreover, the lateral malleolus of *Fruitadens* terminates laterally as an anteroposteriorly broad, bluntly squared off process, rather than being drawn out and tapering laterally as occurs in other ornithischians ([9], [106], [119]; *Scutellosaurus*, MNA P1.175). Although Butler *et al.* [61] listed the morphology of the distal tibia as autapomorphic for *Fruitadens*, we do not consider it autapomorphic here because of the likelihood that the distal tibia of *Heterodontosaurus* has a similar morphology (which is obscured by extensive fusion of the tibia to the proximal tarsals: Santa Luca [11]).

#### Fibula

The left fibula of LACM 120478 has been displaced slightly at the proximal end from its articulation with the tibia (Fig. 14A–E, 15A–D). The fibula is unknown in other specimens, and its distal end is not preserved in articulation with the tibia and astragalocalcaneum of LACM 115727 (Fig. 14I–L). At its proximal end the fibula is expanded anteroposteriorly and compressed transversely, with a gently concave medial surface and a gently convex lateral surface. In lateral view, the proximal surface is gently concave. Beyond about 25% of its length, the shaft of the fibula rapidly narrows to become a very thin rod, and is twisted through nearly 90°. The element is slightly expanded transversely and anteroposteriorly at its distal end where it articulates with the raised platform of the calcaneum and the anterodistal part of the tibia. The distal end of the fibula is convex in lateral view.

The very narrow distal half of the fibula appears to be a heterodontosaurid synapomorphy, being shared with *Heterodontosaurus* [11], although a similar condition occurs in pachycephalosaurs [122]. The distal fibula is relatively broader in other small-bodied ornithischians [118], [121].

#### Astragalocalcaneum

The astragalus and calcaneum are preserved in articulation with the distal tibia in LACM 115727 (Figs. 14I–M, 15E–H) and LACM 120478 (Figs. 14A–D, F, 15A–D). An isolated and partially damaged astragalocalcaneum occurs in LACM 120602 (Fig. 14P, Q). The elements are almost indistinguishably fused with one another, but the point of suture between these two elements is denoted by a line of sediment anteriorly in LACM 120478, and by a sharp anteroposteriorly extending break-in-slope in all specimens, with the calcaneum extending further distally than the astragalus. The astragalus and calcaneum are not fused to the tibia or fibula in any specimen: the suture line with the tibia is still clearly visible in LACM 120478 and LACM 115727, and the fibula is lost in LACM 115727, while only the distal tibiae are preserved in the holotype, and the astragalocalcaneum is isolated in LACM 120478.

In distal view, the astragalus is longer anteroposteriorly than transversely and the calcaneum is transversely narrow and strongly expanded anteroposteriorly (Fig. 14F, M). The astragalus is anteroposteriorly longest at its medial margin (where an anteroposteriorly extending low ridge occurs) and becomes narrower laterally (towards the break-in-slope that marks the point of contact with the calcaneum): it has a distal articular surface that is strongly convex anteroposteriorly. The distal articular surface of the calcaneum is also strongly convex anteroposteriorly. The result is a bird-like distal articular surface that resembles a pulley, with two anteroposteriorly extending ridges (the medial margin of the astragalus and the lateral margin of the calcaneum) separated by an anteroposteriorly convex surface.

The ascending process of the astragalus is unusual for an ornithischian (Figs. 14A, I, P, 15A, E). It has a sub-triangular outline in proximal view, with the apex of the triangle fitting into the notch between the anteromedial sheet and the main body of the tibia. There are two large and prominent foramina on the anterior surface of the ascending process (Figs. 14I, 15E: for): the smaller foramen is positioned medially and the larger one laterally; the lateral foramina opens anterodorsally and is positioned between the proximal process of the calcaneum (see below) and the lateral edge of the ascending process. It is roofed dorsally by a bony connection between the ascending process and the calcaneum. The medial foramen is fully enclosed within the ascending process in LACM 115727, but appears to be open medially (and thus forms a notch in the medial margin of the ascending process) in LACM 120478 (the ascending process is broken in this region in LACM 120602 and so only the margin of the foramen is preserved). The foramina are preserved in three different specimens (LACM 115727, 120478, 120602), and have the same morphology and positions in all three.

Dorsomedial to the foramina, the dorsal margin of the ascending process is a triangular wedge in anterior view, and is separated from the rest of the process by a clear sutural line; moreover, it has a distinct bone surface texture suggesting that it represents a separate ossification (Fig. 15E: dasp). The dorsal part of the ascending process is not preserved in LACM 120602.

The left calcaneum is shaped like a reversed ‘J’ in lateral view, with an elongate proximal process that narrows in anteroposterior width proximally and terminates in a raised articulation for the fibula, and a small concave area posterodistally for articulation with the lateral corner of the tibia. The lateral surface of the calcaneum is strongly concave and depressed. The articular surface for the fibula has an oval outline in proximal view, slopes posterodistally, and is concave (LACM 115727).

The astragalocalcaneum of *Fruitadens* is very similar to that of *Heterodontosaurus*, although extensive fusion and poor preservation complicates anatomical assessment of the latter [11]. In both taxa, the calcaneum and astragalus are indistinguishably fused to one another (the elements are not preserved separately from one another in any known specimen of these taxa) with a pulley-like distal articular surface, the calcaneum is proximodistally elongate with a strongly concave lateral surface, and the astragalus has a very high ascending process. It is unclear whether the apex of the ascending process of *Heterodontosaurus* was formed by a separate ossification, or whether the foramina that occur on the astragalus of *Fruitadens* were present in the former taxon. At present, both of these characters are proposed as autapomorphies of *Fruitadens* but they may eventually prove to be shared with other heterodontosaurids.

The astragalus and calcaneum of other small-bodied ornithischians differ substantially from those of *Fruitadens*. They are typically unfused, the astragalus has a relatively low and laterally offset ascending process, at most a single foramen occurs on the ascending process of the astragalus, the low ascending process does not appear to be formed by a separate ossification, and the calcaneum is relatively short proximodistally [103], [105], [106], [115], [121].

There are similarities between the astragalocalcaneum of *Fruitadens* and that of some “coelophysoid" basal theropods, in which the astragalus and calcaneum are indistinguishably fused to one another [123]. Fusion of the astragalocalcaneum with the distal tibia and fibula, as occurs in *Heterodontosaurus* [11], also occurs in basal theropods [123]. In *Dilophosaurus wetherilli*, the ascending process of the astragalus is also formed by a separate ossification and has two foramina on its anterior surface [124]. Although these similarities are intriguing, current understanding of basal dinosaur phylogeny [114] suggests that they are best interpreted as homoplasies.

#### Pes

The only part of the pes represented in the holotype is a proximally incomplete metatarsal with a crushed shaft, the positional identification of which is uncertain (contra Butler *et al.* [61], who identified it as metatarsal I). At its distal end, the metatarsal is expanded transversely and dorsoventrally to form the condyle. In distal view, this expansion is asymmetrical, being greater on one side than the other. A deep pit occurs on one side (either the lateral or the medial surface) of the condyle.

LACM 120602 contains three metatarsals and three phalanges (Fig. 16). Two of the metatarsals appear to represent metatarsal I from left and right sides (Fig. 16A, B), with the right metatarsal I being damaged at its proximal end. Both are slender, and are expanded dorsally at the proximal end, with a flat lateral surface and a gently convex medial surface. Distally, metatarsal I is expanded both dorsoventrally and transversely to form the condyle, with the transverse expansion being greatest ventrally. No pit occurs on the lateral or medial surfaces of the distal condyle.

The third metatarsal could be metatarsal II, III or IV, but its identity cannot be established with certainty because the proximal end is missing (Fig. 16C, D). It is extremely slender, and the shaft has a nearly square cross section, with flattened dorsal, medial and lateral surfaces. Distally the metatarsal is expanded transversely and dorsoventrally to form the condyle, and there are well-developed ligament pits positioned both medially and laterally, as well as a small pit on the dorsal surface.

The largest of the phalanges is relatively robust, and may represent phalanx III-1 (Fig. 16G, H), based upon comparisons to *Heterodontosaurus* [11]. Its proximal articular surface has an oblong outline, being strongly expanded transversely. The surface of the phalanx is broken dorsally at the proximal end, and so it is unclear if a dorsal projection similar to those present on the equivalent phalanx of *Heterodontosaurus* occurs [11]. Distally, there are well-developed lateral and medial ligament pits, as well as a deep pit on the dorsal surface. A second phalanx is nearly as elongate as the first, but is much more slender, and might represent phalanx I-1 (Fig. 16E). Its proximal articular surface has a nearly square outline and no dorsal projection, and distally there are well-developed lateral and medial ligament pits but no deep pit on the dorsal surface. Finally, the third phalanx is considerably smaller than the other two (Fig. 16F): it has a subtriangular proximal end, the surface of which is saddle shaped, with a small dorsal projection. Distally, lateral and medial ligament pits occur and there is a shallow pit on the dorsal surface.

Comparisons of the pes to other ornithischians are extremely limited, in view of the uncertain positional identifications of the pedal elements of *Fruitadens* and the fragmentary nature of the available material. The morphology of the preserved elements is consistent with that of *Heterodontosaurus* [11], although fusion of the metatarsals to one another is absent. The deep extensor pits on the ends of two of the phalanges is similar to the condition in *Heterodontosaurus* [11] and has previously been identified as a synapomorphy of Heterodontosauridae [59].

### Cranial functional morphology of late-surviving heterodontosaurids

Functional analyses assume that anatomical form is an adaptation to function [125]. Numerous studies have correlated mandibular morphology to loading regime in extant mammals [126]–[128], crocodilians [129], [130], and birds [131], [132]. In contrast, other researchers have demonstrated that physical, phylogenetic, or developmental constraints can lead to a decoupling of form and function [133], as can competing functional requirements that limit the optimization of a feature for a specific function [134]. Although the strength of the link between form and function is uncertain, function can *only* be inferred from form in fossil animals. Quantitative biomechanical methods allow functional hypotheses to be rigorously tested [135]; furthermore, placing biomechanical studies in a phylogenetic context and using independent evidence to corroborate results boosts confidence in functional interpretations [136]. Various biomechanical techniques have been applied to dinosaur mandibles in order to better understand feeding behavior [137], including: lever arm mechanics that estimate bite force and mechanical advantage [138], [139]; free body analyses to predict tensile and compressive stress trajectories [140]; beam modeling [141]; and finite element analysis that predict stress, strain and deformation within the mandible [142]–[144].

Feeding studies in heterodontosaurids have been limited due to the fragmentary nature of most specimens, the exception being *Heterodontosaurus* for which there are well-preserved skulls. The jaw mechanism of *Heterodontosaurus* has been the subject of extensive debate (see Norman *et al.* [14] for a detailed discussion). Proposed jaw mechanisms for *Heterodontosaurus* based on cranial and dental morphology have included: propalinal jaw action [19], [145]; anisosognathus, transverse jaw movements [24]; medial rotation of the dentaries about their long axes [146]; and medial translation (‘inverse wish-boning’) of the dentaries [147]. A detailed reconstruction of the jaw elevator musculature of *Heterodontosaurus* [28] revealed enlarged Group 1 muscles (particularly m. Adductor Mandibulae Externus [mAME]) with long moment arms, suggesting that the jaws of *Heterodontosaurus* were adapted for slow, forceful biting. Further evidence from finite element modeling and tooth microwear supported a jaw mechanism that involved ‘inverse wish-boning’ with some palinal movements [26]. Along with cranial and dental morphology, tooth wear, and lack of tooth replacement, these results suggest that *Heterodontosaurus* used its jaws to process tough vegetation [14], [28], although the presence of enlarged caniniform teeth and strong forelimbs with sharp manual unguals have led some to suggest occasional omnivory [27], [28], [145], [148]. Feeding studies on other heterodontosaurid taxa have been limited to information derived from tooth morphology and wear [23], [24], in which taxa featuring more vertical wear facets (*Abrictosaurus*, *“Lanasaurus"*) are suggested to have employed more orthal jaw movements than those exhibiting oblique wear facets (*Heterodontosaurus*, *Lycorhinus*).

Neither *Fruitadens* nor *Echinodon* preserve the post-dentary bones, and both specimens lack bones of the posterior cranium; in contrast, most of the lower jaw as well as fragments of the jugal, quadratojugal and quadrate (forming the jaw joint) are preserved and articulated in *Tianyulong* [30]. The dentaries and dentitions of *Fruitadens*, *Echinodon* and *Tianyulong* are morphologically similar to each other and differ substantially from those of Early Jurassic heterodontosaurids (see Descriptions and Comparisons above). Recent work on the mandible of *Alligator mississippiensis* has demonstrated that lever arm mechanics can accurately predict simple performance metrics (e.g. reaction force orientation, relative reaction force magnitude, mechanical advantage) because these results are determined by the external geometry of the mandible and the orientations of the forces acting upon it [149]. In contrast, internal stresses and absolute force magnitudes are most accurately modeled using more sophisticated methods, such as finite element analysis, as these metrics are also influenced by the internal shape and material properties of the mandible. We applied simple 2D methods to the mandibles of *Tianyulong* and *Heterotodontasaurus* to obtain basic performance metrics such as mechanical advantage. We suspect that the mandible of *Tianyulong* serves as a functional proxy for that of *Fruitadens* and *Echinodon* due to gross morphological similarities between the mandibles of these taxa, and that our conclusions are applicable to all three heterodontosaurid species.

Unlike *Heterodontosaurus*, *Abrictosaurus*, a specimen possibly referable to *Lycorhinus* (NHMUK C69 [29]), and the undescribed SAM-PK-10488, in which the jaw joint is strongly depressed relative to the occlusal surfaces of the tooth row, the jaw joint of *Tianyulong* is displaced dorsally [30]. If the perpendicular distances between the upper and lower tooth rows to the jaw joint are equal, then the teeth will meet simultaneously along the entire row during jaw closure [150]. If these distances are not equal, the upper and lower teeth will shear past each other and have only a single point of contact that moves anteriorly during jaw closure. Following the method described by Greaves [150], the upper tooth row-to-jaw joint and lower tooth row-to-jaw joint distances are more similar to one another in *Heterodontosaurus* than in *Tianyulong* (Fig. S1). This indicates that the upper and lower tooth rows of *Heterodontosaurus* came into occlusion nearly simultaneously, while the jaws of *Tianyulong* exhibited a more scissor-like action.

When the relative lengths of the moment arms of Group 1 and Group 2 muscle resultants are measured with the jaws at occlusion (see Methods), it is clear that Group 1 muscles have a longer moment arm in *Heterodontosaurus* than in *Tiayulong* (Fig. S2). Herbivorous dinosaur taxa tend to have posteriorly-directed jaw muscle resultants [28], [151]–[153] due to their expanded mAME as evidenced by mediolateral expansion of the posterior cranium (i.e. the adductor chamber). When the jaw joint is depressed, as in *Heterodontosaurus*, the moment arms of the posteriorly directed Group 1 muscles are increased as is the mechanical advantage of the jaws [154]. The Group 1 muscles of *Tianyulong* have shorter moment arms due to the elevated position of the jaw joint; this produces lower mechanical advantage but increases jaw-closing speed [155], [156].

In contrast, Group 2 muscles have a longer moment arm, and thus greater mechanical advantage, in *Tianyulong* than in *Heterodontosaurus*. Living crocodilians, and presumably carnivorous dinosaurs (e.g. *Allosaurus*), possess an enlarged mPT [142], [149]. Optimal sarcomere length, mechanical advantage, and muscle activity patterns, as well as moment arm, suggest that mPT is used for closing the jaws at large gapes in *Alligator* [149]. Although the relative sizes of the jaw adductors in *Tianyulong* are unknown, moment arm lengths suggest that the jaws of *Tianyulong* were adapted for rapid biting at large gape angles, unlike the jaws of *Heterodontosaurus*, which were better suited for strong jaw adduction at small gapes.

*Fruitadens*, *Echinodon* and *Tianyulong* were approximately the same size (body length of ∼70 cm) [30], [37], [39], [61], although the ontogenetic stages of known specimens of *Echinodon* and *Tianyulong* are uncertain. This is considerably smaller than the size of adult *Heterodontosaurus* (body length of ∼1–1.75 metres [29]) and probably other Late Triassic–Early Jurassic heterodontosaurids (i.e. *Lycorhinus*, “*Lanasaurus*") known only from skull material, and possibly *Pisanosaurus* if heterodontosaurid affinities can be demonstrated for this taxon. Thus, it appears that Late Jurassic-Early Cretaceous heterodontosaurids were smaller than most Early Jurassic members of the clade, and displayed less sophisticated skull and dental morphologies. Lack of wear facets and a plesiomorphic tooth morphology suggest that later heterodontosaurids, such as *Fruitadens*, *Echinodon* and *Tianyulong*, used orthal jaw movements and employed simple puncture-crushing to process food. Mandibular functional morphology and the functional analysis presented here further suggest that *Tianyulong* used weak but rapid jaw movements compared to *Heterodontosaurus*. This evidence all points to later heterodontosaurids being ecological generalists, consuming select plant material and possibly insects or other invertebrates [148]. Increased plant consumption favors larger body size in extant lizards [157], strengthening the argument that early, relatively large heterodontosaurids may have been more herbivorous than smaller, later species.

## Discussion

### Monophyly of Heterodontosauridae

The monophyly of Heterodontosauridae has never been seriously questioned, but diagnoses for the clade have generally been based primarily upon cranial characters [12], [13]; but see Sereno [58]. This is because most taxa are known from cranial material only, and, prior to the recent description of *Tianyulong* [30], the only described heterodontosaurid postcranial material was that of *Heterodontosaurus* [11]. Our description of *Fruitadens* provides additional support for the monophyly of Heterodontosauridae from postcranial characters, but slightly weakens support for the clade from cranial characters. Here we discuss the validity of characters that have been proposed by other workers, as well as us [61], to support heterodontosaurid monophyly, and present a revised diagnosis for the clade (above). These characters are discussed within the explicit phylogenetic framework of the cladistic analysis carried out by Butler *et al.* [61].

The presence of three premaxillary teeth has long been considered diagnostic for Heterodontosauridae [59], and indeed appears to be invariant within known members of the clade. This therefore represents a valid synapomorphy of the group, unless heterodontosaurids prove to be closely related to marginocephalians [49], in which case it may prove to be a synapomorphy of Heterodontosauridae+Marginocephalia. The absence of a distinction between the root and the crown of the premaxillary teeth was proposed as a heterodontosaurid synapomorphy by Sereno [59], but the premaxillary crowns are expanded above the root in *Fruitadens* and *Echinodon* [39], potentially invalidating this character (although it may support a more derived clade within Heterodontosauridae). The presence of high-crowned, chisel-shaped ‘cheek’ teeth with denticles restricted to the apical third of the crown and a reduced ‘cingulum’ has been proposed as a heterodontosaurid synapomorphy by several authors [12], [13], [37], but the ‘cheek’ teeth of *Fruitadens* are low, subtriangular, with denticles distributed over more than 50% of the crown and there is a basal ‘cingulum’ (latter also occurring in *Echinodon* [37], [39]). Thus, these characters may also support more derived nodes within Heterodontosauridae, or have undergone reversals in *Fruitadens*, depending on the exact phylogenetic position of *Fruitadens*.

The absence of a dentary caniniform in *Echinodon* [37], [39] and *Abrictosaurus* [19] contradicts suggestions that this is synapomorphic for Heterodontosauridae [12], [13], although its absence could reflect reversals in these taxa. However, regardless of the presence or absence of a caniniform, all heterodontosaurids for which the condition is adequately known possess an arched and recessed diastema between the premaxilla and the maxilla (the condition is uncertain in *Echinodon*, although see Galton [39]). This character may be absent or reduced in *Manidens* [41], but preservation precludes a complete assessment at this stage. Sereno [59] proposed that the absence of denticles in the anterior two dentary teeth and the reduced size of the first dentary tooth (this refers to post-caniniform teeth in those taxa with a caniniform) might be synapomorphic for Heterodontosauridae. Although plausible, the homologies of the anterior dentary teeth in taxa lacking a caniniform (*Echinodon*, *Abrictosaurus*), possessing a caniniform (*Heterodontosaurus*, *Lycorhinus*, *Tianyulong*), and possessing a caniniform and a ‘pre-caniniform’ (*Fruitadens*) requires reinvestigation. Sereno [59] additionally proposed that the wedge-shaped predentary, known only in *Heterodontosaurus* and *Abrictosaurus* and apparently unique amongst ornithischians [14], [19], is also a heterodontosaurid synapomorphy, a view supported by recent phylogenetic analyses [61]. Norman *et al.* [13] noted a number of distinctive cranial features of heterodontosaurids (deep paroccipital process, tall quadrate, elongate posterolateral process of premaxilla), but all of these characters are known with certainly only in *Heterodontosaurus* [14].

Sereno [59] suggested that Heterodontosauridae could be diagnosed by the following postcranial characters: head of the humerus positioned to lateral side in proximal view; elongate manus; metacarpals with blocklike proximal ends; fibula very slender; proximal phalanges of pedal digits II–IV with extensor pits on distal heads. The head of the humerus is indeed positioned laterally in *Fruitadens*, but this does not appear to be the case in *Heterodontosaurus* [11; SAM–PK–K1332]. An elongate manus and blocklike proximal metacarpals occur in basal saurischians [158] and have been reinterpreted as ornithischian plesiomorphies, retained by heterodontosaurids [50], [61]. As discussed above, the fibula is indeed notably slender in *Fruitadens* and *Heterodontosaurus* (although this condition also occurs in pachycephalosaurs), and both taxa possess extensor pits on pedal phalanges. These latter two characters are provisionally accepted as diagnostic of the clade based upon recent phylogenetic analyses [61].

Additional probable heterodontosaurid postcranial synapomorphies suggested by our phylogenetic work [61] are: the presence of a constriction on the proximal surface of the humerus, between the head and the medial tubercle; a ‘rod-like’ (with near parallel sides) fourth trochanter on the femur; and a fused astragalus and calcaneum [61]. Other characters of the distal tibia, particularly the presence of an anteromedial flange, may also prove to be diagnostic of the clade, but cannot be adequately assessed for *Heterodontosaurus* at present.

In summary, the presence in *Fruitadens* and *Echinodon* [37] of a dentition reminiscent of basal ornithischians such as *Lesothosaurus* and *Scutellosaurus* indicates a higher degree of variation in dental morphology within Heterodontosauridae than often appreciated. These dental characters are most plausibly interpreted as retained plesiomorphies in view of the inferred basal positions of *Fruitadens* and *Echinodon* within Heterodontosauridae [61], although they could alternatively be interpreted as reversals. If interpreted as retained plesiomorphies, they weaken craniodental character support for a monophyletic Heterodontosauridae, and suggest that many classic proposed synapomorphies of heterodontosaurids actually diagnose less inclusive clades. By contrast, heterodontosaurid postcranial morphology shows little variation (although limited data is currently available) but is highly unusual within Ornithischia, strongly supporting heterodontosaurid monophyly.

### An overview of heterodontosaurid evolution and biogeography

Heterodontosauridae originated during the Late Triassic, with a single specimen known from the Laguna Colorada Formation (?Norian) of Patagonia, Argentina [6]. Although this specimen is so fragmentary that its heterodontosaurid affinities cannot be considered unquestionable, it is referable to the clade on the basis of current evidence [8]. *Pisanosaurus*, from the upper part of the Ischigualasto Formation (late Carnian), also shares dental characters with Heterodontosauridae [5], [13], but the phylogenetic position of this taxon cannot be resolved at present [8]. Based on present understanding, it seems likely that heterodontosaurids (and ornithischian dinosaurs more generally) were geographically restricted to southern Gondwana during the Late Triassic [7], [8] and that the clade, as for dinosaurs more generally [51], [114], may have originated in this area. The Laguna Colorada specimen, despite its early stratigraphic appearance, already possesses apparently derived heterodontosaurid characters: the maxillary ‘cheek’ teeth are closely packed without spaces between them and are unexpanded above their roots. These similarities led Báez & Marsicano [6] to propose that the Laguna Colorada specimen is phylogenetically close to the Early Jurassic *Heterodontosaurus*, although the specimen has yet to be incorporated into a phylogenetic analysis. If this phylogenetic position proves to be correct, current understanding of heterodontosaurid phylogeny would suggest that heterodontosaurids underwent a phylogenetic and morphological radiation prior to the Triassic/Jurassic boundary. At present, this radiation is not evident in the body fossil record.

The Early Jurassic upper Elliot and Clarens formations of southern Africa currently provide our most complete window on heterodontosaurid morphology and diversity, with the nearly 20 known specimens apparently representing at least four, and possibly more, taxa [29]. These taxa show a range of body sizes: the only known specimen of *Abrictosaurus consors* (NHMUK RU B54), with a femoral length of 78 mm, likely had a body length of around 75–80 cm, similar in size to *Fruitadens* (although the ontogenetic stage of the *Abrictosaurus* specimen is unknown). By contrast, apparently mature specimens of *Heterodontosaurus* (SAM-PK-K1332, NM QR 1788) are inferred to have body lengths between 1–1.75 metres, and a weight ranging between 2 and 10 kg. Other taxa (e.g. *Lycorhinus*) may have been similar in size to *Heterodontosaurus*, although further data is required to establish this. The southern African heterodontosaurids also show a range of craniodental morphologies that potentially imply niche partitioning and slightly different dietary adaptations. In the context of current understanding of heterodontosaurid phylogeny, there appears to have been a trend towards increasing craniodental specializations within the southern African faunal assemblage (see also Pol. *et al.* [41]), with *Heterodontosaurus* showing greater specializations (e.g. more closely packed ‘cheek’ teeth, more extensive tooth-on-tooth wear, loss of the ‘cingulum’) than *Lycorhinus* or *Abrictosaurus*. The relative stratigraphic positions of the southern African heterodontosaurid specimens within the upper Elliot and Clarens formations is extremely poorly resolved, and it is possible that some of this variation could ultimately prove to represent anagenetic evolution rather than multiple coexisting species.

A single heterodontosaurid specimen is known from the Early Jurassic Kayenta Formation (Sinemurian–Pliensbachian) of Arizona [1], [40]. Because this specimen remains undescribed we do not discuss it in detail here. However, it indicates that heterodontosaurids achieved a broader geographical distribution during the Early Jurassic. Although the Kayenta Formation has been relatively well sampled, with numerous specimens of the basal thyreophoran *Scutellosaurus* known [103], [159], only a single heterodontosaurid specimen is currently known, and heterodontosaurids are currently absent from the approximately contemporaneous faunal assemblage of the Lufeng Formation of southern China [46]. This may suggest that heterodontosaurids remained uncommon outside of southern Gondwana during the Early Jurassic. In addition, although currently unknown, ghost lineages indicate that basal heterodontosaurids similar to *Echinodon* and *Fruitadens* must have occurred in the Early Jurassic (see also Pol *et al.* [41]).

Middle Jurassic heterodontosaurids are known only from Argentina [41] and apparently China [30], although the exact stratigraphic position and age of *Tianyulong* is currently unclear. However, numerous tiny ornithischian teeth are known from microvertebrate sites within the Middle Jurassic of Europe [160], [161]. Although generally referred to “Fabrosauridae" or Ornithischia indet., at least some of these teeth may ultimately prove to belong to tiny *Echinodon*- or *Fruitadens*-like heterodontosaurids. These microvertebrate sites hint at a substantial undiscovered diversity of small-bodied ornithischian dinosaurs. A missing diversity of heterodontosaurids is also suggested by ghost lineages that pass through the Middle Jurassic [61].

All currently known Late Jurassic–earliest Cretaceous heterodontosaurid sites (the type localities of *Echinodon*, *Fruitadens*, and possibly *Tianyulong*) are Laurasian, although contemporaneous geological formations in Gondwana are poorly sampled. Thus, heterodontosaurids have been recovered from nearly all continents (with the exception of Australia, India, and Antarctica, all of which have poorly sampled faunas), indicating that they were probably a cosmopolitan group. Although phylogenetic analysis does not suggest that they form a monophyletic grouping [61], these late-surviving and apparently phylogenetically basal heterodontosaurids do show a number of similarities to one another when compared to the currently known Early Jurassic heterodontosaurids, particularly *Heterodontosaurus*. First, *Fruitadens*, *Echinodon*, *Tianyulong* and *Manidens* are all remarkably small, with maximum known body lengths of around 70–80 cm [30], [41], [61] and body masses of <1 kg [39], [61]. Although Early Jurassic heterodontosaurids were also small within the context of Ornithischia as a whole, *Heterodontosaurus* at least reached notably larger body masses [29]. As discussed above, late-surviving heterodontosaurids show relatively unsophisticated craniodental feeding adaptations when compared to the Early Jurassic *Heterodontosaurus*, and are therefore interpreted as more ecologically generalized, and possibly incorporated a greater proportion of animal matter (e.g. small invertebrates) into their diet. These more generalized dietary preferences probably represent retained plesiomorphies, with other early ornithischians also interpreted as omnivores [148].

Although these temporal patterns do not equate into evolutionary trends when viewed in a phylogenetic context [61], they do suggest that those heterodontosaurid lineages that persisted through the Jurassic and into the earliest Cretaceous were small-bodied ecological generalists, and that moderately larger and ecologically more specialized taxa such as *Lycorhinus* and *Heterodontosaurus* were temporally limited to the Early Jurassic (and possibly the Late Triassic, based upon the Laguna Colorada heterodontosaurid). No evidence for trends towards larger body sizes (i.e. Cope's rule) exists when the entirety of heterodontosaurid evolution is examined.

Although the heterodontosaurid fossil record has improved markedly in recent years due to new discoveries and reevaluation of historical taxa, it is still extremely patchy in time and space. A rigorous understanding of heterodontosaurid evolution requires further discoveries, as well as a better-constrained phylogenetic hypothesis. What is clear, however, is that the evolutionary lineage of heterodontosaurids extends for more than 55 million years, making them one of the longest lived of all early dinosaur clades, and implying a substantial diversity that remains largely unsampled by paleontologists.

## Methods

### Micro-CT data

Five of the skull elements described (LACM 115747, left maxilla and right dentary; LACM 128258, left maxilla and left and right dentaries) here were micro-CT scanned at NHMUK by SA Walsh using a HMX-ST CT 225 System (Metris X-Tek, Tring, UK) in February 2009. Data were reconstructed using CT-PRO software version 2.0 (Metris X-Tek). 2000 transverse slices were taken of the left maxilla and left dentary of LACM 115747, and the left maxilla and both dentaries of LACM 128258. Image size and resolution are variable; average voxel size is 0.013 mm. Contrast between fossil material and matrix is excellent. CT data were segmented (to extract bones, teeth, and cavities) and visualized by LBP using Amira 5.3.0 (Visage Imaging, Berlin, Germany; www.amiravis.com). Some additional visualization and generation of rendered images was carried out by RJB using VG Studio MAX 2.0 (Volume Graphics, Heidelberg, Germany). Videos of three of the reconstructions of the cranial elements are available as supplementary material.

### Cranial functional morphology

Lateral reconstructions of the skulls of *Heterodontosaurus* (based on SAM-PK-K1332) and *Tianyulong* [30] were used in the methods described below (Figs. S1 and S2). Due to the incomplete nature of *Fruitadens* and *Echinodon* cranial and mandibular material, the skull of *Tianyulong* was used to represent small-bodied, Middle Jurassic—Early Cretaceous heterodontosaurids in this functional analysis.

To understand how the upper and lower tooth rows came together during jaw closure (Fig. S1), skull reconstructions were scaled to actual size, the upper ‘cheek’ tooth row was set horizontal and the mandible of each taxon was rotated to a gape angle of 15° (measured between the ‘cheek’ tooth rows). Perpendicular distances were measured between the center of the quadrate-articular jaw joint and: 1) a line parallel to the the occusal surface of the maxillary tooth row; 2) a line parallel to the occlusal surface of the dentary ‘cheek’ tooth row, following methods described by Greaves [150].

To compare the relative length of the moment arms for jaw adductor muscles, the skulls were scaled to the same basal skull length and the jaws set at occlusion. 2D vectors (Fig. S2) representing muscle resultants for Group 1 (defined here as the three portions of M. adductor mandibulae externus [mAME], two portions of M. pseudotemporalis [mPST], and M. adductor mandibular posterior [mAMP]) and Group 2 muscles (M. pterygoideus dorsalis [mPTd] and M. pterygoideus ventralis [mPTv]) were mapped onto each skull. Among extant diapsids, Group 1 muscles originate within the supratemporal fossa, on the supratemporal bar and on the anterior surface of the quadrate, and insert on the surangular, coronoid process and mandibular adductor fossa; thus these muscles are directed dorsally and posteriorly relative to the mandible. Group 2 muscles originate on the palate and insert on the posteroventral aspect of the mandible; thus, these muscles are oriented dorsally and anteriorly relative to the mandible [162]. For consistency, Group 1 muscle resultants were drawn between the highest point of the coronoid process and the center of the supratemporal bar on lateral reconstructions of the *Heterodontosaurus* and *Tianyulong* skulls. Group 2 muscle resultants were drawn between the posteroventral margin of the lower jaw and the dorsal surface of the palate. Moment arm lengths for Group 1 and 2 muscles in Figure S2 were divided by mandible length for ease of comparison between the two taxa.

It must be emphasized that these calculations are approximate and involve numerous simplifications. Furthermore, we compare the lengths of the muscle moment arms, not the muscle moments, which account for both moment arm length and muscle force. An accurate functional analysis of the skull of generalized, small-bodied heterodontosaurids (such as *Tianyulong*, *Echinodon* or *Fruitadens*) is not possible at this time due to the fact that the posterior cranium (which serves as the origin for most of the jaw elevator muscles) is missing or deformed in all specimens, making it impossible to estimate muscle size and, therefore, actual muscle or bite force.

### Data archiving

All locality data and taxonomic data and opinions provided by Butler *et al.* [61] and this paper for *Fruitadens haagarorum* have been checked, modified or added to the *Paleobiology Database* (http://paleodb.science.mq.edu.au) by RJB (most original data entered by MT Carrano). *Fruitadens* localities within the *Paleobiology Database* are numbered 53040, 71476 and 92360. Micro-CT data have been reposited with the specimen in the collections of the LACM.

## Supporting Information

Figure S1 **Skull reconstructions of** ***Heterodontosaurus*** **and** ***Tianyulong*** **illustrating the differing nature of contact between upper and lower tooth rows during jaw closure.** Lateral reconstructions of the skulls of *Heterodontosaurus tucki* (A; based on SAM-PK-K1332) and *Tianyulong confuciusi* (B; redrawn from [30], areas of breakage shown in gray). Skulls are scaled to relative size (scale bar equals 1 cm). Using methods developed by Greaves [85], the jaws have been set at a gape angle of 15° between the tooth rows, the perpendicular distance (indicated by red arrows) was measured between the jaw joint and occlusal surfaces of the upper and lower tooth rows. The small difference between these distances (due to a depressed jaw joint) in *Heterodontosaurus* indicate simultaneous occlusion of the upper and lower tooth rows; the larger (relative) difference in *Tianyulong* indicate the jaws closed with a scissor-like action.
(TIF)

Figure S2 **Skull reconstructions of** ***Heterodontosaurus*** **and** ***Tianyulong*** **documenting moment arm lengths for Group 1 and 2 muscles.** *Heterodontosaurus tucki* (A; based on SAM-PK-K1332) and *Tianyulong confuciusi* (B; redrawn from [30], areas of breakage shown in gray). Skulls are scaled to the same size (basal skull length). Black arrows indicate orientation of Group 1 (pointing posterodorsally) and Group 2 (pointing anterodorsally) muscles; see text for explanation of muscle groups and orientation of muscle vectors. Red lines indicate perpendicular moment arms between the jaw joint and Group 1 and 2 muscle vectors (or projections from these vectors, shown by dotted lines). Moment arms were then scaled by mandibular length to produce *relative* moment arm length for each muscle group in both taxa.
(TIF)

Video S1 ***Fruitadens haagororum*** **, LACM 115747 (holotype) left maxilla CT reconstruction.** Maxilla is shown rotating about its longitudinal axis; anterior end is to the left. Elements in the CT reconstructions are colour-coded as follows: maxilla, blue; palatal fragment (vomer?), green; functional teeth, yellow; replacement teeth, orange; internal canals, red. The maxilla and palatal bones have been made transparent in order to better visualize internal anatomy.
(MPG)

Video S2 ***Fruitadens haagororum*** **, LACM 115747 (holotype) right dentary CT reconstruction.** Dentary is shown rotating about its longitudinal axis; anterior end is to the left. Elements in the CT reconstructions are colour-coded as follows: dentary, blue; functional teeth, yellow; replacement teeth, orange; internal canals, red. The dentary bone has been made transparent in order to better visualize its internal anatomy.
(MPG)

Video S3 ***Fruitadens haagororum*** **, LACM 128258 right dentary CT reconstruction.** Dentary is shown rotating about its longitudinal axis; anterior end is to the left. Elements in the CT reconstructions are colour-coded as follows: dentary, blue; functional teeth, yellow; replacement teeth, orange. The dentary bone has been made transparent in order to better visualize its internal anatomy.
(MPG)

Text S1 **Measurements of holotype and referred specimens of** ***Fruitadens haagororum*** **.**
(DOC)
