# Supplementary figures and images for: Anatomy and Cranial Functional Morphology of the Small-Bodied Dinosaur Fruitadens haagarorum from the Upper Jurassic of the USA
Source: PLoS One. 2012 Apr 11;7(4):e31556. doi: 10.1371/journal.pone.0031556 (PMC3324477; doi:10.1371/journal.pone.0031556)

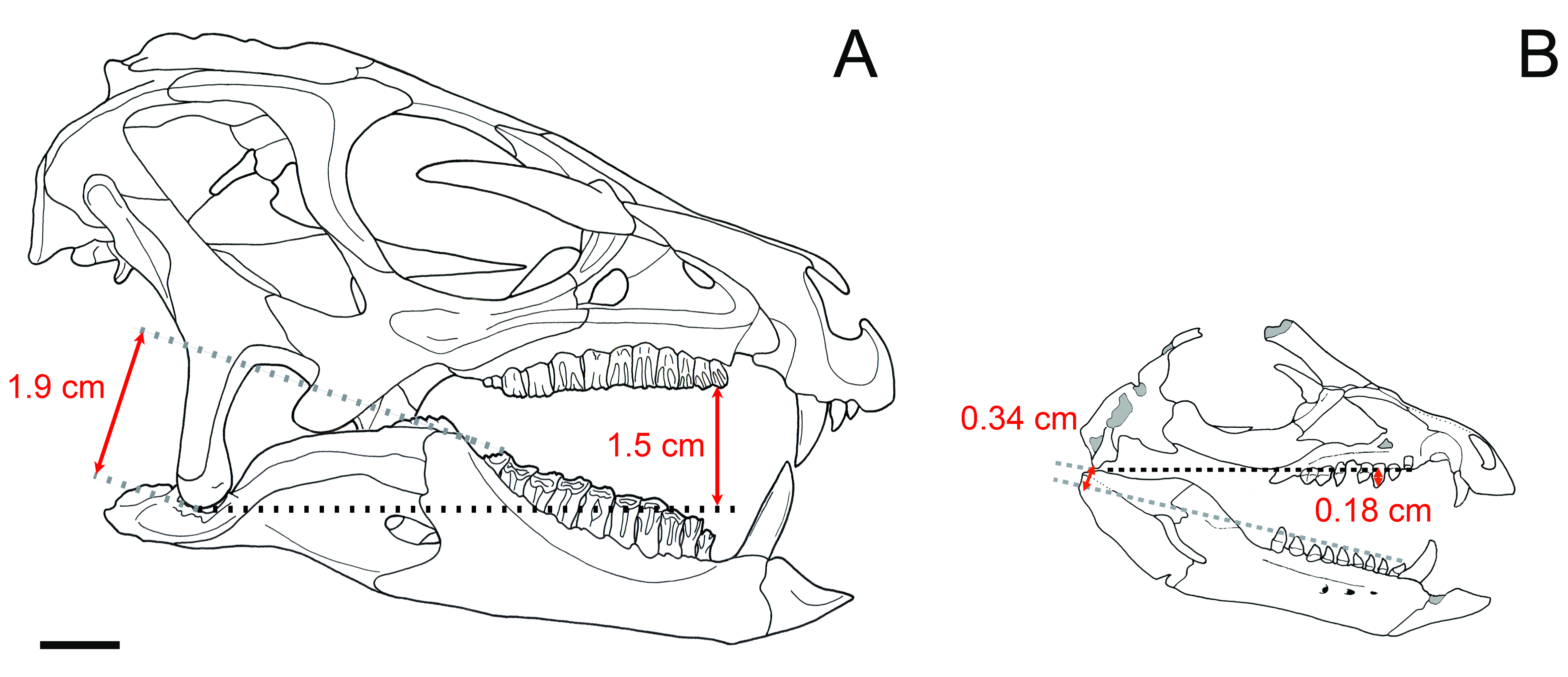

Supplement: Figure S1 — Skull reconstructions of Heterodontosaurus and Tianyulong illustrating the differing nature of contact between upper and lower tooth rows during jaw closure. Lateral reconstructions of the skulls of Heterodontosaurus tucki (A; based on SAM-PK-K1332) and Tianyulong confuciusi (B; redrawn from [30], areas of breakage shown in gray). Skulls are scaled to relative size (scale bar equals 1 cm). Using methods developed by Greaves [85], the jaws have been set at a gape angle of 15° between the tooth rows, the perpendicular distance (indicated by red arrows) was measured between the jaw joint and occlusal surfaces of the upper and lower tooth rows. The small difference between these distances (due to a depressed jaw joint) in Heterodontosaurus indicate simultaneous occlusion of the upper and lower tooth rows; the larger (relative) difference in Tianyulong indicate the jaws closed with a scissor-like action. (TIF) [file pone.0031556.s001.tif]

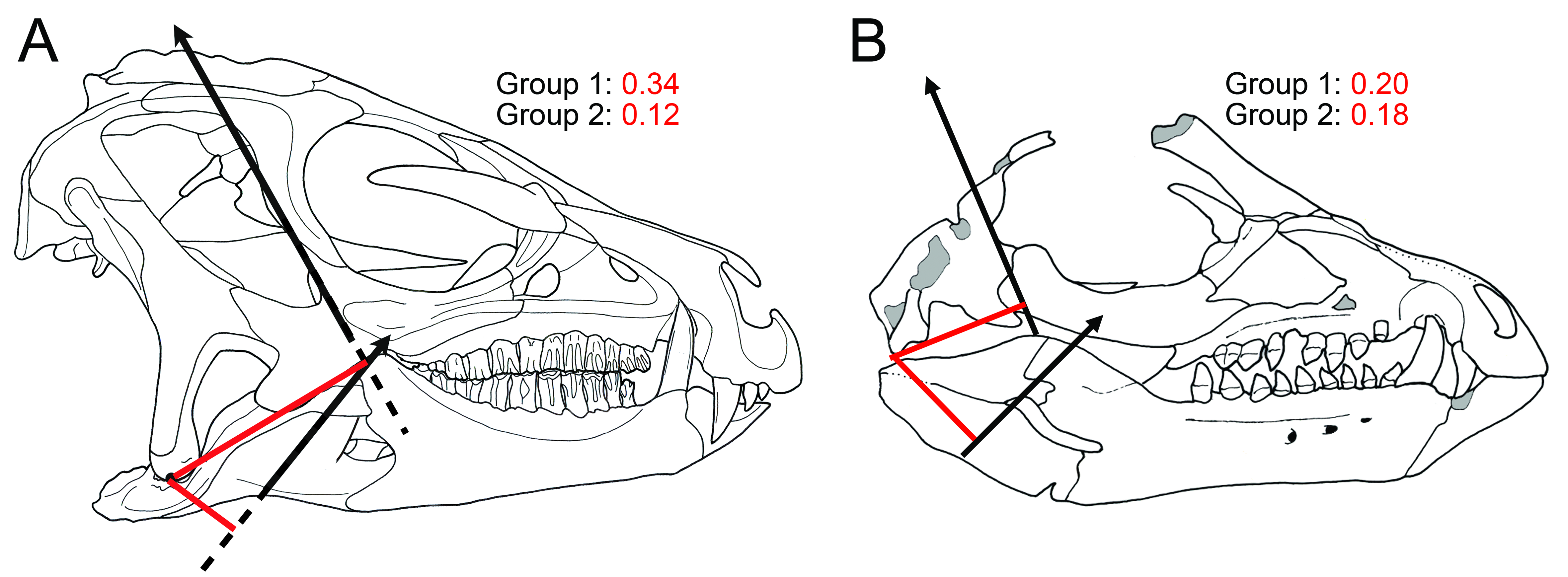

Supplement: Figure S2 — Skull reconstructions of Heterodontosaurus and Tianyulong documenting moment arm lengths for Group 1 and 2 muscles. Heterodontosaurus tucki (A; based on SAM-PK-K1332) and Tianyulong confuciusi (B; redrawn from [30], areas of breakage shown in gray). Skulls are scaled to the same size (basal skull length). Black arrows indicate orientation of Group 1 (pointing posterodorsally) and Group 2 (pointing anterodorsally) muscles; see text for explanation of muscle groups and orientation of muscle vectors. Red lines indicate perpendicular moment arms between the jaw joint and Group 1 and 2 muscle vectors (or projections from these vectors, shown by dotted lines). Moment arms were then scaled by mandibular length to produce relative moment arm length for each muscle group in both taxa. (TIF) [file pone.0031556.s002.tif]
