# Supplementary material for: Anatomy and Cranial Functional Morphology of the Small-Bodied Dinosaur Fruitadens haagarorum from the Upper Jurassic of the USA
Source: PLoS One. 2012 Apr 11;7(4):e31556. doi: 10.1371/journal.pone.0031556 (PMC3324477; doi:10.1371/journal.pone.0031556)
Supplement: Text S1 — Measurements of holotype and referred specimens of Fruitadens haagororum . (DOC) [file pone.0031556.s006.doc]

**Supplementary text (Text S1) to:**

**Anatomy and Cranial Functional Morphology of the Small-Bodied Dinosaur *Fruitadens haagarorum* from the Upper Jurassic of the USA**

**Richard J. Butler^1,2*^, Laura B. Porro^3^, Peter M. Galton^4,5^, Luis M. Chiappe^6^**

**1** Bayerische Staatssammlung für Paläontologie und Geologie, Richard-Wagner-Straße 10, D-80333 Munich, Germany, **2** GeoBio-Center, Ludwig-Maximilians-Universität München, Richard-Wagner-Straße 10, D-80333 Munich, Germany, **3** Department of Organismal Biology and Anatomy, University of Chicago, IL 60637, USA, **4** Professor Emeritus, University of Bridgeport, Bridgeport, CT 06604, USA, **5** 1065 Vintage Drive, Rio Vista, CA 94571, USA, **6** The Dinosaur Institute, Natural History Museum of Los Angeles County, Los Angeles, CA 90007, USA

**Measurements of *Fruitadens haagarorum***

**LACM 115727**

Femur, right, proximal end: mediolateral width = 13.3 mm; anteroposterior width = 10.4 mm

Femur, left, proximal end: mediolateral width = +10 mm; anteroposterior width = 10.5 mm

Tibia, left, proximal end: mediolateral width, proximal end = 9 mm; anteroposterior width = +12 mm

Tibia, left, distal end: mediolateral width = 11.3 mm

Fused astragalus-calcaneum: mediolateral width = 11.3 mm; anteroposterior width = 8 mm

**LACM 115747**

Maxilla, left: preserved length = 22.5 mm

Maxilla, right: length of segment with teeth 1–2 = 7.4 mm; length of segment with teeth 4-6 = 10 mm

Dentary, left: preserved length = 13 mm

Dentary, right: preserved length = 30.5 mm; depth below tooth 7 = 8 mm

Anterior cervical vertebra: centrum length = 8 mm; centrum height (anterior end) = 5.5 mm; centrum width (anterior end) = 5.8 mm; centrum height (posterior end) = 4.8 mm; centrum width (posterior end) = 5.9 mm

Posterior cervical centrum: centrum length = 7.5 mm; centrum height (anterior end) = 5.8 mm; centrum width (anterior end) = 6 mm; centrum height (posterior end) = 5.3 mm; centrum width (posterior end) = 5.8 mm

Anterior dorsal centrum (with ventral flange): centrum length = 7.7 mm; centrum height (anterior end) = 5 mm; centrum width (anterior end) = 5 mm; centrum height (posterior end) = 4.5 mm; centrum width (posterior end) = 5.4 mm

Anterior dorsal centrum 2: centrum length = 7.5 mm; centrum height (anterior end) = 5.6 mm; centrum width (anterior end) = 5 mm; centrum height (posterior end) = 4.9 mm; centrum width (posterior end) = 5.8 mm

Dorsal vertebra 1: centrum length = 9 mm; centrum height (posterior end; anterior end is broken) = 6 mm; centrum width (posterior end) = 5.9 mm; centrum width (anterior end) = 5 mm

Dorsal vertebra 2: centrum length = 8 mm; centrum height (anterior end) = 5.3 mm; centrum width (anterior end) = 5 mm; centrum height (posterior end) = 4.4 mm; centrum width (posterior end) = 5.4 mm

Posterior dorsal centrum 1: centrum length = 9.3 mm; centrum height = 5.5 mm; centrum width = 6.7 mm [Unclear which is anterior and which posterior end]

Posterior dorsal centrum 2: centrum length = 10 mm; centrum height = 5 mm; centrum width = 6.3 mm [Unclear which is anterior and which posterior end]

Sacral vertebra 1: centrum length = 10.1 mm; centrum height (anterior end) = 6 mm; centrum width (anterior end) = 7 mm; centrum height (posterior end) = 5.5 mm; centrum width (posterior end) = 7 mm

Sacral vertebra 2: centrum length = 9.1 mm; centrum height (anterior end) = 5.4 mm; centrum width (anterior end) = 7.5 mm

Sacral vertebra 3: centrum length: 8 mm

Sacral vertebra 4: centrum length: 8 mm; centrum height (posterior end) = 5 mm; centrum width (posterior end) = 7 mm

Sacral vertebra 5: centrum length = 7.9 mm; centrum height (anterior end) = 5 mm; centrum width (anterior end) = 7.5 mm

Sacral vertebra 6: centrum length = 8.1 mm; centrum height (posterior end) = 5 mm; centrum width (posterior end) = 6.4 mm

Caudal vertebra 1: centrum length = 8 mm; centrum height (anterior end) = 5 mm; centrum width (anterior end) = 5.8 mm; centrum height (posterior end) = 5.1 mm; centrum width (posterior end) = 6 mm

Proximal caudal vertebra with transverse process: centrum length = + 8 mm; centrum height (anterior end) = 5.3 mm; centrum width (anterior end) = 5.8 mm [posterior end broken]

Anterior caudal vertebra ‘A’: centrum length = 8.5 mm; centrum height (anterior end) = 5.2 mm; centrum width (anterior end) = 6.5 mm; centrum height (posterior end) = 5.5 mm; centrum width (posterior end) = 5.6 mm

Anterior caudal vertebra ‘B’: centrum length = 10 mm; centrum height (anterior end) = 5.2 mm; centrum width (anterior end) = 5.5 mm; centrum width (posterior end) = 5.4 mm [broken posteriorly so no height possible]

Distal caudal vertebra ‘A’ (well preserved): centrum length = 10.5 mm; centrum height (anterior end) = 4.3 mm; centrum width (anterior end) = 4.6 mm

Distal caudal vertebrae ‘B’ + ‘C’, lengths: 12 mm, 11.5 mm (estimated)

Femur, right, proximal end: mediolateral width = 13.2 mm; anteroposterior width = 10.1 mm

Tibia, left, proximal end: anteroposterior width (incomplete) = 12 mm; mediolateral width = 9.2 mm

Tibia, right and left (measurements identical), distal end: anteroposterior width (including “anteromedial sheet”) = 8 mm; mediolateral width = 10.6 mm

Metatarsal: length = +22 mm

**LACM 120478**

Humerus: length = 36.7 mm; maximum anteroposterior width, proximal end = 4.3 mm; maximum mediolateral width, proximal end = 8 mm; maximum anteroposterior width, distal end = 4.1 mm; maximum mediolateral width, distal end = 6.6 mm; midshaft diameter = 2.7 mm

Femur, left: length of preserved shaft = 42.2 mm; maximum anteroposterior width, distal end = 9 mm; maximum mediolateral width, distal end = 9.6 mm; thickness of shaft proximal to fourth trochanter = 4.2 mm; diameter of hollow medullary cavity at same point = 2.6 mm

Tibia, left: length (tibia and attached astragalus/calcaneum) = 74.1 mm; length (tibia only) = 71.8 mm; maximum anteroposterior width, proximal end = 12.2 mm; maximum mediolateral width, proximal end = 7.3 mm; maximum anteroposterior width, distal end = 7 mm; maximum mediolateral width, distal end = 8.6 mm; midshaft, anteroposterior width = 3.6 mm; midshaft, mediolateral width = 4.3 mm

Fibula, left: length = 61 mm; maximum anteroposterior width, proximal end = 6.9 mm; maximum mediolateral width, proximal end = 2.7 mm; maximum anteroposterior width, distal end = 1.4 mm; maximum mediolateral width, distal end = 2.2 mm; midshaft, diameter = 1 mm

Fused astragalus-calcaneum: transverse width = 9.1 mm; anteroposterior length = 7 mm

**LACM 120602**

Caudal vertebra, length: 9 mm

Fused astragalus-calcaneum: transverse width = 11.5+ mm (incomplete medially); anteroposterior length = 9.2 mm

Metatarsal, 2, 3, or 4: length = 41.5 mm

Metatarsals 1: length = 25 mm (left), 26.5 mm (right)

Phalanx 3-1: length = 13 mm

Phalanx 1-1: length = 12 mm

Third phalanx: length = 8 mm

**LACM 128258**

Premaxilla: preserved anteroposterior length = 7 mm

Maxilla, left: preserved anteroposterior length = 19 mm

Dentary, left: preserved anteroposterior length = 25.5 mm

Dentary, right: preserved anteroposterior length = 24.5 mm

Dorsal vertebra: centrum length = 6.3 mm; centrum height (anterior end) = 3.3 mm; centrum width (anterior end) = 4 mm

Caudal vertebra: centrum length = 6.5 mm

**LACM 128303**

Dentary, left: preserved anteroposterior length = 19 mm
